# Antioxidant properties of drugs used in Type 2 diabetes management: could they contribute to, confound or conceal effects of antioxidant therapy?

**DOI:** 10.1080/13510002.2017.1324381

**Published:** 2017-05-17

**Authors:** Siu Wai Choi, Cyrus K. Ho

**Affiliations:** a Department of Anesthesiology, Queen Mary Hospital, The University of Hong Kong, Pokfulam, Hong Kong SAR; b Faculty of Veterinary and Agricultural Sciences, The University of Melbourne, Melbourne, Australia; c Faculty of Health and Social Sciences, School of Nursing, The Hong Kong Polytechnic University, Kowloon, Hong Kong SAR

**Keywords:** Antioxidants, diabetes, drug management, vitamin C, vitamin E

## Abstract

**Objectives**: This is a narrative review, investigating the antioxidant properties of drugs used in the management of diabetes, and discusses whether these antioxidant effects contribute to, confound, or conceal the effects of antioxidant therapy.

**Methods**: A systematic search for articles reporting trials, or observational studies on the antioxidant effect of drugs used in the treatment of diabetes in humans or animals was performed using Web of Science, PubMed, and Ovid. Data were extracted, including data on a number of subjects, type of treatment (and duration) received, and primary and secondary outcomes. The primary outcomes were reporting on changes in biomarkers of antioxidants concentrations and secondary outcomes were reporting on changes in biomarkers of oxidative stress.

**Results**: Diabetes Mellitus is a disease characterized by increased oxidative stress. It is often accompanied by a spectrum of other metabolic disturbances, including elevated plasma lipids, elevated uric acid, hypertension, endothelial dysfunction, and central obesity. This review shows evidence that some of the drugs in diabetes management have both in vivo and in vitro antioxidant properties through mechanisms such as scavenging free radicals and upregulating antioxidant gene expression.

**Conclusion**: Pharmaceutical agents used in the treatment of type 2 diabetes has been shown to exert an antioxidant effect..

## Introduction

The number of people worldwide with diabetes mellitus (DM) has increased from 30 million to 180 million since 1985 [1–3]. This increase is anticipated to continue, with the fastest increases seen in Asia [1,2,4]. Over 90% of diabetes is Type 2 DM, which is also associated with dyslipidemia, hypertension, and elevated plasma uric acid [3,5], which can lead to long term micro- and macro-vascular complications.

Owing to their various metabolic problems, most Type 2 DM subjects are commonly treated by a ‘polypharmacy’ of oral hypoglycemic agents, statins, fibrates, and anti-hypertensive drugs of various types [6]. Some of these have been reported to have pleiotropic effects and antioxidant properties [7–9]. Oxidative stress is increased in DM, and there is often a depletion of body stores of ascorbic acid (vitamin C), which is an important dietary-derived antioxidant. Antioxidant supplementation has been suggested as a potentially beneficial adjunct therapy [10,11]. In this article, we review the literature in relation to antioxidant effects of drugs used in the management of diabetes, and discuss results of antioxidant supplementation studies in DM patients from the perspective of the possible confounding or concealing influence of drug-induced antioxidant effects.

## Type 2 DM and its management: a brief overview

Type 2 DM is a state of continuous or intermittent hyperglycemia caused by a relative deficiency of insulin. Tissues are insulin resistant and there may also be β-cell dysfunction or failure [12]. Hypoglycemic agents are aimed at improving insulin secretion and action, and limiting or slowing absorption of glucose in the gastrointestinal tract [13]. These drugs are taken orally, and may be combined with insulin injections for those patients who fail to respond acceptably to any of the oral hypoglycemic agents alone (Table 1).

**Table 1.** Oral hypoglycemic agents used in type 2 DM.

Oral hypoglycemic drugs (Brand name)	Chemical name and formula	Chemical structure	Mechanism of primary action	Typical dosage/blood levels	C_max_ & AUC of drug
Metformin (Glucophage, Glucophage XR, Glumetza, Fortamet, Riomet)	3-(diaminomethylidene)-1,1-dimethylguanidine C_4_H_11_N_5_		Increases glucose transport across the cell membrane in skeletal muscle. Acts only in the presence of endogenous insulin	500–850 mg daily. Maximum daily dose 2550 mg.	C_max_ = 1713.54 ± 508.75 ng/ml AUC = 9284.88 ± 2155.57 ng.h/ml [14]
Glipizide (Glucotrol)	N-[2-[4-(cyclohexylcarbamoylsulfamoyl)phenyl]ethyl]-5-methylpyrazine-2-carboxamide C_21_H_27_N_5_O_4_S		Binds to ATP-sensitive potassium-channel receptors on pancreatic cell surface, decreasing potassium conductance, and causing depolarization of membrane. This stimulates calcium ion influx through voltage-sensitive calcium channels, inducing secretion, or exocytosis, of insulin.	5 mg administered 30 min before meals. Maximum dose 40 mg daily.	C_max_ = 523 ± 60 ng/ml AUC = 1897ng.h/ml [15]
Glyburide/ Glibenclamide (Micronase, Diabeta, Glynase Prestab)	5-chloro-N-[2-[4-(cyclohexylcarbamoylsulfamoyl)phenyl]ethyl]-2-methoxy benzamide C_23_H_28_ClN_3_O_5_S		Same as glipizide.	2.5–5 mg daily of regular tablets or 1.5–3 mg daily of micronized tablets. Maximum dose 1.25–20 mg of regular tablets and 0.75–12 mg of micronized tablets.	C_max_ = 147.1 ± 23.6 ng/ml AUC = 525.7 ± 28.1 ng.h/ml [16]
Tolbutamide (Orinase)	1-butyl-3-(4-methylphenyl)sulfonylurea C_12_H_18_N_2_O_3_S		Same as glipizide.	500 mg.	C_max_ = 63 ± 11 μg/ml AUC = 721 ± 94 μg.h/ml [17]
Nateglinide (Starlix)	(2S)-3-phenyl-2-[(4-propan-2-ylcyclohexanecarbonyl)amino]propanoic acid C_19_H_27_NO_3_		Stimulates pancreas to produce insulin (similar to the sulfonylureas). Appears to have a faster onset and a shorter duration of action than sulfonylureas. May prevent the rapid, transient rise in blood glucose that occurs immediately following a meal.	60 or 120 mg three times daily.	C_max_ = 3.09 ± 1.64 μg/ml AUC = 6.93 ± 1.99 μg.h/ml [18]
Repaglinide (Prandin)	2-ethoxy-4-[2-[[(1S)-3-methyl-1-(2-piperidin-1-ylphenyl)butyl]amino]-2 -oxoethyl]benzoic acid C_27_H_36_N_2_O_4_		Stimulates insulin production. Rapid onset and short duration of action.	Immediately before a meal.	C_max_ = 30.96 ± 9.06 μg/l AUC = 36.03 ± 6.00 μg.h/l [19]
Rosiglitazone maleate (Avandia)	5-[[4-[2-[methyl(pyridin-2-yl)amino]ethoxy]phenyl]methyl]-1,3-thiazolidine-2,4-dione C_18_H_19_N_3_O_3_S		Attaches to insulin receptors on cells throughout the body increasing insulin sensitivity.	4–8 mg daily.	C_max_ = 724.3 ± 135.7 ng/ml AUC = 4024 ± 956 ng.h/ml [20]
Pioglitazones (Actos)	5-[[4-[2-(5-ethylpyridin-2-yl)ethoxy]phenyl]methyl]-1,3-thiazolidine-2 ,4-dioneC_18_H_19_N_3_O_3_S C_19_H_20_N_2_O_3_S		Same as rosiglitazone.	15–45 mg daily.	C_max_= 932 ± 335 ng/ml AUC = 8.61 ± 3.66 mg.h/l [21]
Miglitol (Glycet)	1-(2-hydroxyethyl)-2-(hydroxymethyl)piperidine-3,4,5-triol C_8_H_17_NO_5_		Inhibitors of intestinal α-glucosidase enzymes, resulting in delayed breakdown of carbohydrates and delayed glucose absorption, reducing postprandial hyperglycemia.	25–100 mg three times before meals.	C_max_ = 1883,9 ± 912.4 μg/l AUC = 7592.7 ± 4207.4 μg.h/l [22]
Acarbose (Precose) Alpha-glucosidase inhibitors	(3R,4R,5S,6R)-5-[(2R,3R,4R,5S,6R)-5-[(2R,3R,4S,5S,6R)-3,4-dihydroxy-6-methyl-5-[[(1S,4R,5S,6S)-4,5,6-trihydroxy-3-(hydroxymethyl)cyclohex-2-en-1-yl]amino]oxan-2-yl]oxy-3,4-dihydroxy-6-(hydroxymethyl)oxan-2-yl]o xy-6-(hydroxymethyl)oxane-2,3,4-triol C_25_H_43_NO_18_		Slows down the actions of α-amylase and α -glucosidase enzymes.	25–100 mg three times daily.	Data not available.
Sitagliptin	(3R)-3-amino-1-[3-(trifluoromethyl)-6,8-dihydro-5H-[1,2,4]triazolo[4,3-a]pyrazin-7-yl]-4-(2,4,5-trifluorophenyl)butan-1-one C_16_H_15_F_6_N_5_O		Inhibits dipeptidylpeptidase-4, increases insulin secretion and lowers glucagon secretion.	100 mg once daily.	C_max_ = 706nMAUC = 7.13μM.h [23]

Type 2 DM is often accompanied by a spectrum of other metabolic disturbances, including elevated plasma total and low density lipoprotein cholesterol (TC and LDL-C), low high density lipoprotein cholesterol (HDL-C), elevated triglycerides (Tg), elevated uric acid, hypertension, endothelial dysfunction, central obesity, and elevated inflammatory biomarkers (high sensitivity C-reactive protein (hsCRP) and inflammatory cytokines such as interleukin 6 (IL-6) [24]. The drugs most commonly used to treat dyslipidemia and hypertension in Type 2 DM patients are presented in Table 2.

**Table 2.** Lipid-lowering and anti-hypertensive drugs commonly used in type 2 DM management.

	Chemical name and formula	Chemical structure	Mechanism of primary action	Typical dosage/blood levels	C_max_ & AUC of drug
**Lipid- lowering drugs (Brand name)**					
Lovastatin (Mevacor, Lipivas, etc.)	[(1S,3R,7S,8S,8aR)-8-[2-[(2R,4R)-4-hydroxy-6-oxooxan-2-yl]ethyl]-3,7-dimethyl-1,2,3,7,8,8a-hexahydronaphthalen-1-yl] (2S)-2-methylbutanoate C_24_H_36_O_5_		Readily hydrolyzes *in vivo* to the corresponding β-hydroxyacid, a potent inhibitor of HMG-CoA reductase. Stimulates the production of low-density lipoprotein receptors in the liver.	10–80 mg daily.	C_max_ = 5.57 ± 0.61 ng/ml AUC = 39.45 ± 3.23 ng.h/ml [25]
Pravastatin (Mevastatin, Selectin, Elisor, etc.)	(3R,5R)-7-[(1S,2S,6S,8S,8aR)-6-hydroxy-2-methyl-8-[(2S)-2-methylbutanoyl]oxy-1,2,6,7,8,8a-hexahydronaphthalen-1-yl]-3,5-dihydroxyheptanoic acid C_23_H_36_O_7_		Inhibits HMG-CoA reductase and hepatic synthesis of VLDL-C, reducing circulating cholesterol and LDL-C.	40 mg daily. Maximum dose is 80 mg.	C_max_ = 115.8 ± 77.5 ng/ml AUC = 259.0 ± 133.4 ng.h/ml [26]
Simvastatin (Lipex, Cholestat, Zocor, etc.)	[(1S,3R,7S,8S,8aR)-8-[2-[(2R,4R)-4-hydroxy-6-oxooxan-2-yl]ethyl]-3,7-dimethyl-1,2,3,7,8,8a-hexahydronaphthalen-1-yl] 2,2-dimethylbutanoate C_25_H_38_O_5_		The six-membered lactone ring of simvastatin is hydrolyzed *in vivo* to generate mevinolinic acid, an active metabolite structurally similar to HMG-CoA. Once hydrolyzed, simvastatin competes with HMG-CoA for HMG-CoA reductase.	5–80 mg daily.	C_max_ = 16.3 ± 81.4 ng/ml AUC = 93.5 ± 70.1 ng.h/ml [27]
Atorvastatin (Lipitor, Tulip, Torvast, etc.)	(3R,5R)-7-[2-(4-fluorophenyl)-3-phenyl-4-(phenylcarbamoyl)-5-propan-2-ylpyrrol-1-yl]-3,5-dihydroxyheptanoic acid C_33_H_35_FN_2_O_5_		Selectively and competitively inhibits HMG-CoA reductase.	20–40 mg daily.	C_max_ = 44.7 ± 61.3 ng/ml AUC = 164.7 ± 58.1 ng.h/ml [27]
Fluvastatin (Lescol, Cranoc, Canef, etc.)	(E,3S,5R)-7-[3-(4-fluorophenyl)-1-propan-2-ylindol-2-yl]-3,5-dihydroxyhept-6-enoic acid C_24_H_26_FNO_4_		Selectively and competitively inhibits HMG-CoA reductase.	20–80 mg daily.	C_max_ = 60.81 ± 38.26 ng/ml AUC = 246.97 ± 141.95 ng.h/ml [28]
Rosuvastatin (Crestor)	3*R*,5*S*,6*E*)-7-[4-(4-fluorophenyl)-2-(*N*-methylmethanesulfonamido)-6-(propan-2-yl)pyrimidin-5-yl]-3,5-dihydroxyhept-6-enoic acid C_22_H_28_FN_3_O_6_S		Competitive inhibitor of HMG-CoA reductase.	10–40 mg daily.	C_max_ = 12.3 ± 7.0 ng/ml AUC = 124 ± 71 ng.h/ml [29]
Gemfibrozil (Lipozid, Lopid, Bolutol, Gemlipid, etc.)	5-(2,5-dimethylphenoxy)-2,2-dimethylpentanoic acid C_15_H_22_O_3_		Increases the activity of extrahepatic LPL, increasing lipoprotein triglyceride lipolysis. Inhibits synthesis and increases the clearance of apolipoprotein B.	600 mg twice a day. Maximum daily dose 1200 mg.	C_max_ = 18.57 ± 6.61 μg/ml AUC = 75.19 ± 26.30 μg.h/ml [28]
Colestipol (Cholestabyl, Colestid)	N'-(2-aminoethyl)-N-[2-(2-aminoethylamino)ethyl]ethane-1,2-diamine; 2-(chloromethyl)oxirane C_11_H_28_ClN_5_O		A non-absorbed, lipid-lowering polymer. Binds bile acids in the intestine, impeding their reabsorption. This upregulates cholesterol 7-(alpha)-hydroxylase, increasing the conversion of cholesterol to bile acids.	2–16 grams (tablets) or 5–30 grams for the granules per day.	Data not available.
Fenofibrate (Antara, Lipidil, Procetofen, etc.)	propan-2-yl 2-[4-(4-chlorobenzoyl)phenoxy]-2-methylpropanoate C_20_H_21_ClO_4_		Activates Peroxisome proliferator-activated receptor alpha, increasing lipolysis and elimination of triglyceride-rich particles from plasma by activating lipoprotein lipase and reducing production of apolipoprotein C-III.	40–120 mg per day.	C_max_ = 9.00 μg/ml AUC = 56.4 μg.h/ml [30]
Niacin (Diacin, Daskil, Nicoside, Simcor, etc.)	pyridine-3-carboxylic acid C_6_H_5_NO_2_		Binds to nicotinate D-ribonucleotide pyrophsopate phosphoribosyltransferase, nicotinic acid phosphoribosyltransferase, nicotinate N-methyltransferase and the niacin receptor. Decreases esterification of hepatic Tg.	Recommended daily allowance range from 2 to 18 mg a daily.	C_max_ = 4.61 ± 1.30 μg/ml AUC = 11.6 ± 1.24μg.h/ml [31]
**Anti-hypertensive drugs (Brand name)**					
Ramipril (Acovil, Vesdil, Delix, etc.)	(2S,3aS,6aS)-1-[(2S)-2-[[(2S)-1-ethoxy-1-oxo-4-phenylbutan-2-yl]amino]propanoyl]-3,3a,4,5,6,6a-hexahydro-2H-cyclopenta[d]pyrrole-2-carboxylic acid C_23_H_32_N_2_O_5_		Competes with angiotensin I for binding at the angiotensin-converting enzyme (ACE), blocking the conversion of angiotensin I to angiotensin II.	2.5–20 mg daily.	C_max_ = 16.6 ± 13.4 ng/ml AUC = 39.5 ± 31.2 ng.h/ml [32]
Lisinopril (Zestril, Linopril, Lisipril, etc.)	(2S)-1-[(2S)-6-amino-2-[[(2S)-1-hydroxy-1-oxo-4-phenylbutan-2-yl]amino]hexanoyl]pyrrolidine-2-carboxylic acid C_21_H_31_N_3_O_5_		Competes with angiotensin I for its binding site on ACE.	10–40 mg daily.	C_max_ = 79.8 ± 39.4 μg/ml AUC = 992.8 ± 520.5 μg.h/ml [33]
Amlodipine (Norvasc, Amvaz, Lotrel, Lipinox, etc.)	O3-ethyl O5-methyl 2-(2-aminoethoxymethyl)-4-(2-chlorophenyl)-6-methyl-1,4-dihydropyridine-3,5-dicarboxylate C_20_H_25_ClN_2_O_5_		Inhibits the influx of extracellular calcium across the myocardial and vascular smooth muscle cell membranes, causing dilation of coronary and systemic arteries.	5–10 mg once daily.	C_max_ = 19.8 ± 6.7 ng/ml AUC = 359.2 ± 129.5 ng.h/ml [34]
Chlorthalidone (Thalitone, Oradil, Zambesil, Hygroton, etc.)	2-chloro-5-(1-hydroxy-3-oxo-2H-isoindol-1-yl)benzenesulfonamide C_14_H_11_ClN_2_O_4_S		Indirectly increases potassium excretion via the sodium-potassium exchange mechanism by increasing the delivery of sodium to the distal renal tubule.	25–100 mg once daily.	C_max_ = 3.15 ± 0.52 μg/ml AUC = 5.55 ± 1.58 μg.h/ml [35]
Losartan (Cozaar, Lacidipine, Hyzaar, etc.)	[2-butyl-5-chloro-3-[[4-[2-(2H-tetrazol-5-yl)phenyl]phenyl]methyl]imidazol-4-yl]methanol C_22_H_23_ClN_6_O		Interferes with binding of angiotensin II to angiotensin II AT_1_-receptor in vascular smooth muscle and adrenal gland.	50–100 mg daily.	C_max_ = 351.0 ± 167.57 ng/ml AUC = 497.76 ± 171.90 ng.h/ml [36]
Captopril (Lopril, Acepril, Apopirl, Capoten, etc.)	(2S)-1-[(2S)-2-methyl-3-sulfanylpropanoyl]pyrrolidine-2-carboxylic acid C_9_H_15_NO_3_S		Competes with angiotensin I for binding at the ACE, blocking the conversion of angiotensin I to angiotensin II.	25–150 mg daily. Maximum dose is 450 mg.	C_max_ = 234 ng/ml AUC = 15.1μg.h/ml [37]
Furosemide (Diurin, Lasix, Nadis, Salurex, Uritol, etc.)	4-chloro-2-(furan-2-ylmethylamino)-5-sulfamoylbenzoic acid C_12_H_11_ClN_2_O_5_S		Inhibits the reabsorption of sodium and chloride in ascending limb of the loop of Henle, increases the urinary excretion of sodium, chloride, water, potassium, hydrogen, calcium, magnesium, ammonium, and phosphate.	40 mg twice daily.	C_max_ = 1087 ± 262 ng/ml AUC = N/A [38]
Metoprolol (Lopressor, Toprol, Prelis, Betaloc, etc.)	1-[4-(2-methoxyethyl)phenoxy]-3-(propan-2-ylamino)propan-2-ol C_15_H_25_NO_3_		Competes with adrenergic neurotransmitters (catecholamines) for binding at beta(1)-adrenergic receptors in heart and vascular smooth muscle resulting in decrease in heart rate, cardiac output, and blood pressure.	100–450 mg daily.	C_max_ = 114.62 ± 32.92 μg/l AUC = 2096.45 ± 791.79 μg.h/l [39]
Nifedipine (Adalat, Afeditab, Procardia, Glopir, Oxcord, etc.)	dimethyl 2,6-dimethyl-4-(2-nitrophenyl)-1,4-dihydropyridine-3,5-dicarboxylate C_17_H_18_N_2_O_6_		Inhibits influx of extracellular calcium through myocardial and vascular membrane pores by physically plugging the channel causing dilation of the coronary and systemic arteries.	10–20 mg daily.	C_max_ = 36.55 ± 6.76 ng/ml AUC = 347.06 ± 51.61 ng.h/ml [40]
Atenolol (Atcardil, Corotenol, Tenormin, Xaten, etc.)	2-[4-[2-hydroxy-3-(propan-2-ylamino)propoxy]phenyl]acetamide C_14_H_22_N_2_O_3_		Competes for binding at beta(1)-adrenergic receptors in heart and vascular smooth muscle, inhibiting sympathetic stimulation, resulting in lowered systolic and diastolic blood pressure.	50–100 mg once daily.	C_max_ = 709 ± 221 ng/ml AUC = 6120 ± 2171 ng.h/ml [41]

## Antioxidant effects of drugs used in the management of Type 2 DM

As noted, there is a polypharmacy armory for the management of patients with DM, and although many patients may be managed using dietary strategies alone, many patients with diabetes will be on a combination of several of these drugs. Many of these drugs exhibit pleiotropic effects, that is, beneficial effects separate from their primary action. As summarized in Table 3, *in vitro* studies, using the Ferric Reducing Antioxidant Power (FRAP) Assay, have shown that some drugs do show antioxidant properties (our unpublished data). While the FRAP assay was originally developed to investigate antioxidant power in biological samples, such as plasma and urine, it has since been applied to foods and health products, and since it is a test for the intrinsic chemical antioxidant properties of an agent, it was applied to drugs in this instance [42,43].

**Table 3.** FRAP value of some drugs used in the management of Type 2 DM.

		FRAP per tablet/capsule (μmol)			
	Mol. Mass	Water	HCl	NaOH	Ethanol
Hypoglycemic agent					
Metformin HCl (Brand A), 500 mg	165.6	–	–	–	–
Metformin HCl (Brand B), 500 mg	165.6	–	–	–	–
Chlopropamide, 250mg	276.7	–	–	–	–
Gliclazide, 80 mg	323.4	–	–	740	543
Glipizide, 5 mg	445.5	–	–	–	–
Tolbutamide, 500 mg	270.4	–	–	–	–
Pioglitazone, 2 mg	356.4	–	–	202	182
Rosiglitazone, 2 mg	357.4	–	–	19	9
Glibenclamide, 5 mg	494	–	–	–	–
Lipid- lowering agent					
Fluvastatin sodium, 40 mg	433.5	–	–	–	–
Simvastatin, 10 mg	418.6	23	26	–	14
Pravastatin sodium, 10 mg	424.5	–	–	–	–
Lovastatin, 20 mg	404.5	–	–	–	–
Acipimox, 250 mg	154.1	–	–	–	–
Bezafibrate, 400 mg	361.8	–	–	–	–
Gemfibrozil, 900 mg	250.3	–	–	–	–
Fenofibrate, 300 mg	260.8	–	–	–	–
Probucol, 250 mg	519.6	–	–	–	223
Rosuvastatin, 10 mg	481.5	–	–	–	–
Nicotinic acid, 500 mg	123.1	–	–	–	100
Atorvastatin calcium, 10 mg	1209.4	–	–	–	–
Antihypertensive agent					
Metoprolol, 50 mg	267.4	–	–	22	42
Doxazosin mesylate, 5 mg	451.5	–	–	–	16
Tritace, 5 mg	416.5	–	–	13	–
Valsartan, 80 mg	435.5	–	–	427	1962
Amlodipine, 5 mg	408.9	–	–	27	22

FRAP: ferric reducing antioxidant power; HCl: hydrochloric acid; NaOH: sodium hydroxide; –: no detectable FRAP.

Studies by other investigators have shown that these drugs used in the management of DM can improve antioxidant status and ameliorate oxidative stress in cell culture, animal, and human trials (Table 4) [44,45].

**Table 4.** Antioxidant effects of drugs commonly used in treatment of diabetes: (a) Oral hypoglycemic drugs; (b) Lipid-lowering and anti-hypertensive drugs.

Subject characteristics	Study description/duration of treatment	Outcome/biomarkers investigated	Results/comments
Type 2 DM patients [46]	Glipizide alone (15 mg/day) [*n* = 74] Glipizide (15 mg/day) with Aralia root bark extract (2.7 g/day) [*n* = 74] 8 weeks	HbA1c, fasting plasma glucose, 2h-postprandial plasma glucose, TC, and LDL-C	Both treatments decreased HbA1c (from 7.3 to 6.4% in combination group and from 6.9% to 6.5% in glipizide alone), compared to baseline (*p* < 0.05).
Type 2 DM patients [47]	Glibenclamide (5 mg/day) [*n* = 15] Gliclazide (80 mg/day) [*n* = 15] 12 weeks	Plasma lipid peroxides and plasma TRAP	Gliclazide significantly lowered plasma lipid peroxides (from 18.2 ± 3.9 to 13.3 ± 3.8 μmol/l), increased plasma TRAP (from 997.8 ± 132.5 to 1155.6 ± 143 μmol/l), compared to baseline control (*p* = 0.0001).
Type 2 DM patients Healthy, age, and gender-matched controls [48]	(1) Gliclazide 80–240 mg/day [*n* = 33] (2) Glibenclamide 10–15 mg/day [*n* = 30] (3) Metformin 1500 mg/day [*n* = 29] (4) Healthy, age, and gender-matched controls [*n* = 28] All Type 2 DM subjects already on antidiabetic agents (gliclazide, glibenclamide, or metformin) for ≥ 6 months before enrollment into study	TAS and plasma levels of o-Lab	In group 1, both the o-LAb level (236.6mIU/ml) and TAS (1.39 mmol/l) were similar to those of control group. o-Lab level (435.7mIU/ml, *p* < 0.05) was highest and TAS (0.85 mmol/l, *p* < 0.05) was lowest in group 3, compared with control.
Newly diagnosed Type 2 DM subjects [49]	Metformin or gliclazide uptitrated until good glycemic control was achieved, (up to 2550 mg/day for metformin and of 240 mg/day for gliclazide) [*n* = 26] 12 weeks	Urinary 8-iso-PGF2α, plasma vitamins A and E	Metformin significantly decreased 8-iso-PGF2α (from 708 to 589 pg/mg creatinine, *p* < 0.001), while no changes in these parameters were observed with gliclazide treatment. Gliclazide treatment did not affect plasma concentrations of vitamin A and E. Metformin increased plasma vitamin A from 1.84 to 2.23 µmol/l and vitamin E from 12.8 to 18.2 µmol/l, (no p value given and unsure if vitamin E value was lipid-standardized).
Type 2 DM patients, non-smokers, non-drinkers, free of chronic disease [50]	Gliclazide [*n* = 16] Metformin [*n* = 15] Diet treatment [*n* = 15] Time frame and dose unknown	Erythrocyte GPx, GST, catalase, MDA, and GSH	GPx, GST, and catalase activities were significantly higher, MDA levels were significantly lower in both gliclazide and metformin treatment groups compared with diet group (*p* < 0.05). No significant difference in antioxidant enzyme activities and levels of MDA between the drug treatment groups. No differences were seen in GSH between the three groups.
Lean polycystic ovary syndrome (BMI <25 kg/m^2^) subjects and age- weight-matched healthy controls DM, hypertension and renal dysfunction were exclusion criteria [51]	Rrosiglitazone (4 mg/day) [*n* = 25] Metformin (850 mg twice/day) [*n* = 25] Age-weight-matched healthy controls [*n* = 35] 12 weeks	Serum MDA and TAS	TAS increased (from 0.95 to 1.21 mmol/l, *p* < 0.005) and MDA decreased (from 7.46 to 4.02 nmol/ml, *p* < 0.001) after rosiglitazone, but no changes were seen in these parameters after metformin. After treatment, MDA concentrations significantly higher in the metformin group compared with the rosiglitazone group (*p* < 0.05).
Healthy subjects aged 31–65 years with BMI > 27 kg/m^2^ Randomized, double-blind study [52]	Rosiglitazone (4 mg/day) [*n* = 20] Placebo [*n* = 20] 6 months	Plasma peroxides	Compared with placebo, plasma peroxides decreased significantly after 6 months of rosiglitazone treatment (−15%).
Type 2 DM patients on a stable dose of oral hypoglycemic drugs for at least 6 months prior to the study, with HbA1c 7–10% and with postprandial plasma glucose > 8 mmol/l [53]	Repaglinide and diet treatment [*n* = 21] Repaglinide and metformin [*n* = 25] Diet treatment [*n* = 13] Metformin alone, for 2 months [*n* = 16]	TAS, SOD, HbA1c	TAS increased from 20.62 ± 0.9 to 24.31 ± 1.4 μgH_2_O_2_/ml/min (*p* < 0.05) in repaglinide-treated group; and also serum SOD activity increased from 208.1 ± 10.7 to 237.9 ± 10.6 U/l (*p* < 0.0004). Both showed a significant difference compared to control group. HbA1c level was decreased in repaglinide-treated group from 8.6 ± 0.97 to 8.0 ± 1.0%, compared to control group (8.3 ± 1.4–8.2 ± 1.2%, *p* = 0.01).
Newly diagnosed Type 2 DM patients [54]	Pioglitazone (30 mg/day) [*n* = 30] Metformin (1000 mg/day) [*n* = 50] No medication (lifestyle modification) for 3 months [*n* = 49]	AOPP, AGE, FRAP, enzymatic activities of PON, LCAT, LPL	Significant decreases in AOPP (from 154.20 ± 3.75 to 125.45 ± 4.08 μmol/l, *p* < 0.001) and AGE (from 68.62 ± 0.52 to 59.58 ± 0.91%, *p* < 0.001) observed in patients taking pioglitazone, whereas FRAP (from 1051.30 ± 25.54 to 1183.27 ± 46.67 μmol/l, *p* = 0.003) and activities of both PON (from 37.07 ± 2.19 to 48.25 ± 3.00 U/l, *p* < 0.001) and LPL (from 2.27 ± 0.17 to 5.03 ± 0.19 U/ml, *p* < 0.001) increased significantly. No changes were seen in control group, except a decrease in FRAP
15 Type 2 DM patients and 20 healthy, age-, and sex-matched controls [55]	Metformin (1700mg/day) [*n* = 15] Placebo [*n* = 20] 3 months	MDA, SOD, ascorbic acid, α-tocopherol, AOPP, AGE, NAG.	Increases in serum NAG activity (*p* < 0.05) and plasma MDA were found 1 month after metformin treatment, whereas plasma MDA (*p* < 0.001), ascorbic acid (*p* < 0.01), and the α-tocopherol/ (cholesterol + triglyceride) ratio (*p* < 0.001) increased significantly after metformin treatment.
Type 2 DM patients [56]	Metformin (850–2000mg/day) [*n* = 110] Placebo [*n* = 98] 24 weeks	HbA1c, intracellular ROS generation, AOPP, AGE.	Compared to baseline, HbA1c, AOPP, and AGE decreased significantly from 8.7 ± 1.4 to 6.9 ± 0.75% (*p* < 0.05), from 179.64 ± 13.6 to 120.65 ± 10.5 μmol/l (*p* < 0.001), and from 107 ± 10.4 to 78 ± 7.6 pmol/l (*p* < 0.05), respectively. Reduction in ROS generation was seen in white blood cells in patients in metformin group.
87 Type 2 DM patients and 45 healthy controls [57]	Dietary treatment only (newly diagnosed DM patients) [*n* = 42] Glucose-lowering treatment with average dose of metformin at 1000–1500 mg/day (diagnosed for more than 5 years) [*n* = 45] Healthy controls [*n* = 45] 3 months	Serum LDL-C oxidation, HbA1c	HbA1c level and oxidized-LDL-C/LDL-C ratio decreased from 8.37 ± 0.36 to 7.36 ± 0.59 μmol/l (*p* < 0.001) and from 0.082 to 0.06 (*p* < 0.001), respectively, after metformin.
Newly diagnosed Type 2 DM patients [58]	Metformin (1000 mg/day) [*n* = 50] No medication (lifestyle medication) [*n* = 49] 3 months	AOPP, AGE, FRAP, PON, LCAT, LPL.	Compared to baseline, a significant reduction in AOPP (from 137.52 ± 25.59 to 118.45 ± 38.42 μmol/l, *p* < 0.001), and AGE (from 69.28 ± 4.58, 64.31 ± 8.64%, *p* = 0.002) seen after metformin treatment. FRAP (from 1060.67 ± 226.69 to 1347.80 ± 251.40 μmol/l, *p* < 0.001) and PON (from 29.85 ± 23.18 to 37.86 ± 27.60 U/l, *p* = 0.012) increased significantly. Significant differences between metformin and lifestyle modification were observed only in AOPP (*p* = 0.007), FRAP, and AGE (*p* < 0.001).
Subject characteristics/test material	Study description	Outcome/biomarkers investigated	Results/comments
*Simvastatin*			
Hypercholesterolemic subjects (with serum cholesterol > 200 mg/dl and proven vascular disease: coronary, carotid, peripheral arterial disease) [59]	Cross-over trial with two groups Group 1: simvastatin 10–40 mg/day, titrated to achieve ≥20% reduction in cholesterol after 60 days Group 2: same simvastatin treatment + vitamin E 600 mg/day with meals [*n* = 43] 2 months	Plasma vitamin E, 8-hour urinary 8-iso-PGF2α and oxLDL	Urinary 8-iso-PGF2α and ox-LDL was significantly decreased by simvastatin alone (both *p* < 0.05), but the addition of vitamin E produced no further reduction in urinary 8-iso-PGF2α or ox-LDL. Inverse correlation between 8-iso-PGF2α and vitamin E was seen (r^2^ = 0.024, *p* = 0.318).
*Simvastatin*			
Male subjects with previously untreated hypercholesterolemia with fasting serum cholesterol between 6.0–8.0 mmol/l and fasting Tg < 3.0 mmol/l [60]	Subjects randomly allocated to habitual diet [*n* = 60] or dietary treatment group [*n* = 60]. Each group further randomized to receive simvastatin (20 mg/day) or placebo 12 weeks	Serum α-tocopherol, β-carotene, and ascorbic acid. Serum LDL-C fraction used for the determination of diene conjugation and total peroxyl radical trapping antioxidant potential. Antioxidant potential of isolated LDL-C samples was determined.	Simvastatin treatment did not affect serum ascorbic acid, but decreased serum α-tocopherol by 16.2% and β-carotene by 19.5% and total peroxyl radical trapping potential of serum LDL-C by 16.9%. Relative antioxidant power of LDL-C preparations (LDL-C TRAP/mmol of LDL-C) increased by 17.4 Note: The crude value of α-tocopherol was not corrected for the cholesterol levels.
Hypercholesterolemic patients [61]	Three-phase protocol (1) Washout, without statin treatment (basal) for 1 month (2) Simvastatin (20 mg/day) for 2 months (3) Simvastatin (20 mg/day) + α-tocopherol (400UI/day) for 2 months [*n* = 25]	Plasma α-tocopherol, lycopene, retinol, β-carotene, nitrotyrosine, and GSH	The concentration of retinol increased by 37% in group 2. No difference seen for other lipid-soluble antioxidants in groups 2 and 3. Simvastatin alone and simvastatin taken with α-tocopherol decreased plasma nitrotyrosine to the same degree (*p* < 0.05 compared to control group). GSH concentrations were not affected by any treatment.
*Simvastatin*			
Patients with LDL > 130 mg/dl [62]	Simvastatin (20 or 40 mg/day) [*n* = 72] 8 weeks	Oxidative DNA damage in lymphocytes	Simvastatin treatment decreased DNA damage, reduction of 10.5–8.8% in tail DNA, 55.5–44.5 µm in tail length, and 7.5–5.2 in tail moment (tail DNA: *r* = 0.586; tail length: *r* = 0.580; tail moment: *r* = 0.576 respectively, *p* < 0.001 for all when compared to baseline.
Subjects with LDL-C ≥ 130 mg/dl Subjects free of hepato- and renal disorders, DM, history of CVD and had not taken any cholesterol-lowering agent during the preceding 6 months [63]	Simvastatin (20–40 mg/day) administered Randomly assigned to 20 mg/day [*n* = 39] or 40 mg/day [*n* = 37] 8 weeks	Ox-LDL, TRAP, and plasma antioxidant vitamins (retinol, α-tocopherol, γ-tocopherol, coenzyme Q10 and carotenoids)	In all subjects, simvastatin treatment improved plasma antioxidant status as assessed by TRAP (increased from 1.20 to 1.23 µM, *p* < 0.05). Ox-LDL was significantly decreased from 63.4 to 41.5 U/l (*p* < 0.0001). Significant increases in the levels of retinol (*p* < 0.001), α-tocopherol (*p* < 0.001) and γ-tocopherol (*p* < 0.005) seen. Coenzyme Q10 and carotenoids remained unchanged.
*Simvastatin*			
Type 2 DM patients with dyslipidemia, Type 2 DM patients without dyslipidemia and healthy control subjects [64]	Observational study Dyslipidemic Type 2 DM patients on 20 mg simvastatin daily for an average of 2 years, [*n* = 9] Dyslipidemic Type 2 DM patients not on simvastatin, [*n* = 14] Non-dyslipidemic Type 2 DM patients controls [*n* = 11] Healthy controls [*n* = 18]	Oxidative DNA damage in leukocytes; plasma MDA; TAR; HbA1c; PON activity; CRP	TAR and PON activity was significantly increased in patients under simvastatin treatment when compared to those without simvastatin treatment, whereas MDA, CRP, and DNA damages were found decreased significantly compared to patients without simvastatin treatment and patients without dyslipidemia
*Atorvastatin*			
Normocholesterolemic Type 2 DM patients All subjects were (treated with oral glucose-lowering agents and/or insulin) [65]	Atorvastatin (10 mg/day) [*n* = 10] Placebo [*n* = 9] 3 months	LDL oxidizability (dienes production), plasma α-tocopherol	Dienes production lower in the atorvastatin group (487 vs. 538 nmol, *p* = 0.012) compared to placebo. Plasma α-tocopherol was decreased in the atorvastatin group (8.67 vs. 10.55 µg/ml plasma, *p *= 0.036) compared to the placebo group.
*Atorvastatin*			
Subjects without clinical evidence of carotid artery disease, active liver disease and renal insufficiency and with LDL-C ≥ 130 mg/dl [66]	Atorvastatin (10 mg/day) [*n* = 35] 12 weeks	Plasma levels of protein-bound chlorotyrosine, nitrotyrosine, dityrosine, and orthotyrosine, plasma CRP.	Decrease in chlorotyrosine, dityrosine, and nitrotyrosine of 30, 32, and 25%, respectively, *p* < 0.02 each) after atorvastatin. Non-significant decreases in CRP and orthotyrosine seen (11 and 9%, respectively, *p *> 0.01 each).
Hyperlipidemic Type 2 DM patients with LDL-C >130 mg/dl [67]	Atorvastatin (10 mg/day), [*n* = 110] 24 weeks	Percentage change in free radical scavenger enzymes, SOD, GSH, GST, GPx, plasma MDA.	Compared to baseline, SOD, and GSH activities increased significantly (*p* < 0.001), whereas plasma MDA level significantly decreased (*p* < 0.001). No changes were seen in other biomarkers.
Type 2 DM patients without macroangiopathy [68]	Atorvastatin (10 mg/day), [*n* = 15] Vitamin C (2 g/day), [*n* = 13] No treatment control, [*n* = 14] 4 weeks	Serum IL-6, TNF-α, sVCAM-1, CRP, and ADMA.	Serum IL-6 (from 2.27 ± 0.55 to 1.36 ± 0.19 pg/ml), TNF-*α* (from 2.51 ± 0.54 to 1.62 ± 0.18 pg/ml), sVCAM-1 (from 484.7 ± 31.2 to 363.5 ± 13.2 ng/ml), CRP (from 3.84 ± 1.16 to 2.34 ± 0.71 mg/l) and ADMA (from 1.21 ± 0.18 to 0.72 ± 0.08 µmol/l) showed significant decreases after atorvastatin treatment (*p* < 0.05 compared to baseline), while no changes were seen in the other two groups.
Hypercholesterolemic patients (not taking statins or antioxidants) free of cardiovascular disease, liver disease, and renal insufficiency. Healthy controls [69]	Subjects were randomized to A) diet only [*n* = 15] B) diet + atorvastatin (10 mg/day) [*n* = 15] C) healthy control subjects [*n* = 20] Followed-up for 30 days after treatment	Urinary 8-iso-PGF2α and plasma vitamin E and concentrations	A reduction of 8-iso-PGF2α (−37.1%, *p* < 0.01) and ox-LDL (−58.9%, *p* < 0.01), increase of vitamin E (42%, *p* < 0.01) and a reduction of cholesterol (−24.9%, *p* < 0.01) seen after 30 days. In vitro study showed that atorvastatin dose-dependently inhibited LDL-C oxidation and 8-iso-PGF2α formation.
Type 2 DM patients [70]	Atorvastatin (10 mg/day) [*n* = 23] Curcumin (120 mg twice/day) [*n* = 23] Placebo [*n* = 21] 8 weeks	Plasma MDA	Plasma MDA was found decreased significantly from 3.46 ± 0.51 to 2.16 ± 0.17 nmol/ml, compared to baseline (*p* < 0.01) in those taking atorvastatin.
*Fenofibrate*			
Type 2 DM patients with no complications or dyslipidemia [71]	Fenofibrate (200 mg/day) only, [*n* = 18] Coenzyme Q10 (200 mg/day) only, [*n* = 19] Fenofibrate (200 mg/day) and coenzyme Q10 (200 mg/day), [*n* = 19] Placebo, [*n* = 18] 12 weeks	HbA1c and plasma F2 isoprostanes	Fenofibrate did not result in significant changes in any markers.
*Fenofibrate*			
Patients with combined dyslipidemia, with cholesterol in the range of 5.0–7.8 mmol/l, Tg ≥ 1.7 mmol/l 40% of the subjects had history of Type 2 DM, 65% of hypertension, 25% of CHD, 15% of previous stroke [72]	Fenofibrate (300 mg/day) [*n* = 20] 8 weeks to 3 months	Plasma MDA, TAS, GPx, conjugated diene formation	Fenofibrate treatment resulted in significant reduction conjugated diene from 33.7 to 19.7 µmol/l (-42%) (*p* < 0.0001). A tendency toward lower TAS (from 1.72 to 1.62 mmol/l, *p* = 0.061) and a similarly non-significant trend to decreased MDA production (from 1.95 to 1.81 µmol/l, *p* = 0.199) were noted. Significant increase in GPx activity (80%, from 5.8 at the baseline to 10.45 U/ml) was seen.
*Lovastatin*			
Type 2 DM patients with dyslipidemia [73]	Policosanol (10 mg/day); [*n* = 16] Lovastatin (20 mg/day); [*n* = 16] 8 weeks treatment after 4 weeks on a cholesterol-lowering diet	Plasma TBARS	Lovastatin decreased plasma TBARS by 11.5% compared to baseline (*p* < 0.001).
*Lovastatin*			
Hemodialysis patients [74]	Lovastatin (20 mg/day), [*n* = 30] Hypolipemic diet [*n* = 28] Control group [*n* = 13] 6 months treatment	Serum TAS and 8-OHdG	A significant decrease in 8-OHdG from 15.6 to 12.5 ng/ml (*p* = 0.04), and a significant increase in the TAS level from 1.28 to 1.37 mmol/l (*p* = 0.011) was seen in lovastatin-treated group. TAS decreased in the hypolipemic diet- treated group from 1.55 to 1.45 mmol/l (*p* = 0.007).
*Fluvastatin*			
Type 2 DM patients with hyperlipidemia [75]	Fluvastatin (20 mg/day), [*n* = 6] Controls, [*n* = 6] 12 weeks.	Plasma LDL size, LHPO, TBARS, oxysterols	LHPO (from 43.3 ± 13.1 to 14.4 ± 5.1 nmol/mgLDL), TBARS (from 8.83 ± 1.11 to 4.27 ± 0.65 nmol/ml), Total oxysterols (from 61.25 ± 8.32 to 38.95 ± 6.57 ng/ml) decreased significantly after 12 weeks in treatment group compared to baseline (*p* < 0.05).
Gemfibrozil			
Type 2 DM patients with hypertriglyceridemia [76]	Gemfibrozil (1200 mg/day) [*n* = 56] 3 months	Serum PON activity	Serum PON activity increased from 100.2 to 118.7 U/I, *p* < 0.001.
*Combination*			
Type 2 DM patients with hypercholesterolemia (LDL cholesterol level > 100 mg/dl) [77]	Cross-over study [*n* = 45] Simvastatin alone (20 mg/day) Ramipril (5–10 mg/day) and simvastatin Ramipril alone Each treatment for 2 months with a washout period of 2 months.	Plasma MDA	Plasma MDA levels were decreased by 4 ± 7% (*P* = 0.026) and by 25 ± 4% (*p* < 0.001) in simvastatin alone or simvastatin with ramipril, respectively, when compared to baseline.
Type 2 DM patients with dyslipidemia [78]	Cross-over trial Simvastatin (20 mg/day), [*n* = 10]; Fenofibrate (200 mg/day), [*n* = 10] Treatment for 3 months; 2 month wash-out period, patients crossed over on other treatment for 3 months Healthy controls [*n* = 24]	HbA1c, uric acid, homocysteine, plasma MDA, SOD, GSH, serum ascorbic acid, serum α-tocopherol, NAG activity	Uric acid decreased from 370 ± 90 to 313 ± 107 μmol/l (*p* < 0.01), MDA decreased from 2.78 ± 0.40 to 2.36 ± 0.36 μmol/l, and homocysteine increased from 11.9 ± 2.8 to 16.0 ± 4.5 μmol/l (*p* < 0.001), in patients treated with fenofibrate, this was not found in those taking simvastatin. GSH, ascorbic acid, and NAG increased from 14.4 ± 5.0 to 11.4 ± 5.4 μmol/l (*p* < 0.01), 56.4 ± 23.2 to 75.0 ± 20.6 μmol/l (*p* < 0.001), 23.4 ± 5.7 to 31.4 ± 7.3 U/I (*p* < 0.001), respectively, in patients treated with simvastatin but not with fenofibrate. α-tocopherol decreased in both treatments from 18.3 ± 5.5 to 14.9 ± 3.9 mg/l (*p* < 0.001) in simvastatin and from 18.8 ± 5.2 to 14.7 ± 3.6 mg/l in fenofibrate.
*Combination*			
Type 2 DM patients [79]	Rosiglitazone (4 mg/day) in addition to patients’ current therapy for 12 weeks, followed by the addition of fenofibrate (200 mg /day) for another 12 weeks, [*n* = 40] 24 weeks	HbA1c, Uric acid	Uric acid decreased significantly from 4.78 ± 1.1 to 4.41 ± 1.1 mg/dl (*p* = 0.001), whereas HbA1c decreased from 9.95 ± 9.7 to 8.85 ± 8.5% compared to baseline. Addition of fenofibrate resulted in a significantly lower uric acid concentration when compared to rosiglitazone alone (4.41 ± 1.1 mg/dl vs. 3.86 ± 0.99 mg/dl, *p* = 0.001)
Patients requiring statin treatment but not taking any antioxidants or statins 2 months prior to the study [80]	Simvastatin (20 mg/day) [*n* = 17] Pravastatin (40 mg/day) [*n* = 18] 6 weeks	Plasma α- and γ-tocopherol	Plasma α- tocopherol levels were equally decreased by pravastatin and simvastatin (-17.5 µg/ml, *p* = 0.0002 and -12.2 µg/ml, respectively, *p* = 0.006) when compared to baseline. Pravastatin did not significantly affect plasma *γ*- tocopherol levels, simvastatin produced a significant increase (22 µg/ml, *p* = 0.009)
Subjects with metabolic syndrome, healthy control subjects [81]	Simvastatin [*n* = 16] Atorvastatin [*n* = 14] Metabolic syndrome patients not on statin [*n* = 82] Healthy control subjects [*n* = 80] Dosage not specified. >6-month statin treatment	Retrospective analysis of oxidative stress assessed by serum levels of 8-OHdG and vitamin E.	Statin-treated subjects had higher levels of vitamin E (5.24 vs.4.61 µmol/mmol cholesterol, *p* = 0.02) and decreased levels of 8-OHdH (2.16 vs3.68 ng/ml, *p* < 0.01) compared with subjects not on statin. Statin treated subjects had similar vitamin E and 8-OHdG levels as control subjects.
*Combination*			
Type 2 DM subjects with marked hypertriglyceridemia. All diabetic patients were treated with either metformin or a combination of metformin and a sulfonylurea to achieve HbA1c of < 9% before the study. 6-week washout/run-in period for lipid-lowering medications before subjects were randomly assigned to a treatment group [82]	(1) Atorvastatin 20 mg/day, [*n* = 19]; (2) Micronized fenofibrate 200 mg/day, [*n* = 19] 6 weeks treatment period	Plasma oxLDL, 8-iso-PGF2α , ApoB and ApoA-I, E-selectin	Atorvastatin treatment significantly reduced plasma ApoB (−43.2%, *p* < 0.0001), as did treatment with fenofibrate (-9.9%, *p* = 0.01). OxLDL was significantly reduced in atorvastatin group (−38.4%, *p* < 0.0001). A tendency toward decreased 8-iso-PGF2α levels in atorvastatin group (−62.1%, *p* = 0.06) and decreased levels of oxLDL in fenofibrate group (-8.8%, *p* = 0.09) were observed. Under fenofibrate treatment, the % change in plasma levels of oxLDL was significantly and directly correlated with LDL-C (*r* = 0.60, *p* = 0.006) and with the % change in soluble E-selectin levels (*r* = 0.64, *p* = 0.003). Such associations were not significant in atorvastatin group.
*Combination*			
Type 2 DM patients with serum LDL levels > 100 mg/dl [83]	Atorvastatin, 20 mg/day, [*n* = 31]; Rosuvastatin, 10 mg/day, [*n* = 31] 3 months	TAC, TOS, LOOH	TAC increased significantly (from 0.87 ± 0.17 to 0.97 ± 0.13 mmol Trolox equiv/l, *p* = 0.007) in atorvastatin group. No changes seen in other markers or in patients taking rosuvastatin.
*Valsartan*			
Hypertensive patients (BP > 140/90 mmHg) with mild diabetes or impaired glucose tolerance (HbA1c < 9%, requiring treatment by diet alone or an oral hypoglycemic) [84]	Valsartan (40–80 mg/day) – initial dose 40 mg/day, increased to 80 mg/day if the BP was still 140/90 mmHg after 1 month [*n* = 26] 3 months	8-OHdG and 8-isoprostanes	Urinary 8-OHdG decreased significantly from 12.12 to 8.07 ng/mg. creatinine (*p* = 0.001). The decrease in 8-OHdG following valsartan treatment is independent of the amelioration in %HbA1c (or glucose metabolism). In contrast, concentrations of 8-isoprostanes did not alter significantly after 3 months of treatment (283.5 vs. 302.0 pg/mg. creatinine post-treatment, *p* = 0.559).
*Losartan*			
Type 2 DM patients with nephropathy [36]	Losartan (100 mg/day) [*n* = 678]; Matching placebo [*n* = 664] Mean follow-up duration of 3.4 years	Serum uric acid, HbA1c	Losartan reduced serum uric acid compared to placebo (*p* < 0.001). HbA1c level was not affected by losartan treatment.
*Niacin*			
Type 2 DM patients with reduced HDL cholesterol levels and metabolic syndrome [85]	Extended-release niacin from 500 mg/day for 3 months, with escalation to 1000 mg/day every month; [*n* = 15] Healthy controls [*n* = 10]	Endothelial superoxide production, NADPH oxidase activity, and lipid oxidation of HDL as measured by electrophoresis mobility and MDA concentration.	Decreased endothelial superoxide production (*p* = 0.004), NADPH oxidase activity (*p* = 0.04), myeloperoxidase activity (*p* = 0.02) and myeloperoxidase content (*p* = 0.01) were observed in subjects taking niacin, compared to placebo. Reduced lipid peroxidation of HDL seen in subjects taking niacin only when evaluated using electrophoresis mobility (*p* = 0.02) but not by measuring MDA concentration (*p* = 0.41), compared to placebo group.
*Others*			
Type 2 DM patients with hypertension, BP > 160/95 mmHg [37]	Captopril, [*n* = 16] Enalapril, [*n* = 16] Doses given according to patient requirement 12 weeks.	Plasma TBARS	Both captopril and enalapril treatments gave rise to significant decreases in TBARS levels from 2.35 to 1.46 nmol MDA (*p* < 0.05) and from 2.44 to 1.72 nmol MDA (*p* < 0.01), respectively.
Non-obese, normotensive patients with Type 2 DM [86]	Cross-over study Enalapril, 10 mg/day or 30 mg/day continuous-release Nifedipine, for 4 weeks, followed by 2-week washout period then crossed over to the other treatment for 4 weeks [*n* = 24]	LDL oxidizability, HbA1c level	Enalapril reduced LDL oxidation significantly (*p* = 0.001), whereas nifedipine increased LDL oxidation (*p* < 0.05). HbA1c level was not affected by either treatment.
*Others*			
Hypertensive patients Healthy control subjects [87]	(1) α-blocker: doxazosin (4 mg/day), [*n* = 20]; (2) β-blocker: metoprolol (100 mg/day,) [*n* = 22]; (3) ACE inhibitor: ramipril (5 mg/day), [*n* = 20]; (4) angiotensin II antagonist: valsartan (80 mg/day), [*n* = 20]; (5) calcium channel blocker: amlodipine (10 mg/day), [*n* = 20]; Healthy controls [*n* = 51] 3 months	Erythrocyte MDA and SOD	Erythrocyte MDA decreased (*p* < 0.001) and SOD increased (*p* < 0.05) in groups 3 and 4 when compared to baseline. No obvious effect on oxidative stress was noted in other groups.
Type 2 DM patients with hypertension [88]	Carvedilol (initiated at 6.25 mg twice daily up to a maximum of 25 mg twice/day), [*n* = 16]; Metoprolol (initiated at 50 mg twice/day up to a maximum of 200 mg twice/day), [*n* = 18] In addition to patients’ current anti-hypertensive medications 5 months.	Oxidized LDL, HbA1c, and CRP, 8-isoprostane	No significant differences were observed between either groups and compared to baseline.
*Others*			
Type 2 DM patients (aged 43–78 years) with nephropathy whose systolic arterial 200 > BP > 130 mmHg and who were absent of marked hyperglycemia (HbA1c < 8%) Patients with a history of vascular events or revascularization, severe diabetic retinopathy, current smokers were excluded. None of the patients have received ARB, diuretics, xanthine oxidase inhibitors, anti-inflammatory drugs,	Subjects were randomly assigned to (1) Candesartan 8 mg/day, [*n* = 11] or valsartan 80 mg/day, [*n* = 22] (2) Trichlormethiazide 2 mg/day [*n* = 33] 8 weeks	Urinary levels of 8-epi-PGF2α and 8-OHdG, normalized with creatinine.	Treatment with either candesartan or valsartan for 8 weeks reduced the levels of urinary 8-epi-PGF2α and 8-OHdG, whereas levels were not altered with trichlormethiazide treatment. Significant correlation was observed between the reduction of the urinary 8-epi-PGF2α and 8-OHdG and those of the nephropathy (urinary albumin and type IV collagen). There was no significant difference in the studied biomarkers between the candesartan and the valsartan group.
*Others*			
or drugs with antioxidant property within 1 month before the study. Majority of the patients had been treated with ACE inhibitor or calcium channel blockers for ≥1 year [89]			

The antioxidant effects of statins have come under intense research [90–92]. Atorvastatin has decreased superoxide production in human endothelial cells exposed to high glucose, and also protected these cells from hydrogen peroxide mediated damage [93,94]. In human studies, statins have been shown to protect lymphocytic DNA from oxidative damage, decrease concentrations of oxidized-LDL, plasma concentrations of protein-bound tyrosines, urinary F_2_-isoprostanes as well as to increase concentrations of an erythrocyte antioxidant enzyme, superoxide dismutase (SOD) [59,62,66,69,81,87,93,95].

Although both *in vitro* and *in vivo* studies provide evidence of the antioxidant properties of statins, conflict exists with regard to the effect of statins on plasma tocopherols (vitamin E). Cangemi et al. [69] found in a retrospective study that subjects with metabolic syndrome on statin therapy (simvastatin or atorvastatin) for 6 months or more had significantly higher concentrations of plasma vitamin E (*p* = 0.02) and lower concentrations of plasma 8-hydroxy-2'-deoxyguanosine (8-OHdG) (*p* < 0.01) compared to those subjects with metabolic syndrome not treated with statins [81]. The statin-treated group had similar plasma vitamin E and 8-OHdG concentrations as the healthy control group. While in Jula et al.’s (2002) study of hyper-cholesterolemic subjects, 12-week treatment with simvastatin had no effect on serum ascorbic acid, but decreased serum α-tocopherol by 16.2% and β-carotene by 19.5% [60]. Plasma α-tocopherol was also decreased in normocholesterolemic subjects given atorvastatin for 3 months [60,65]. It is noted here though that Jula’s and Oranje’s work did not lipid-standardize the measurements of the lipid-soluble vitamins, and therefore, the decreases seen may simply be due to the lipid-lowering effect of the statins [60,65]. Plasma α-tocopherol was not significantly changed after 2 months of simvastatin treatment in hyper-cholesterolemic subjects in De Caterina’s [59] study, and additional supplementation with vitamin E did not enhance the antioxidant effect seen when simvastatin was taken alone.

The biguanide metformin is one of the most widely prescribed oral hypoglycemic medications [96,97]. *In vivo* studies have shown that metformin was able to scavenge hydroxyl radicals, and to reduce the production of ROS in bovine aortic endothelial cells, though the results in these studies are mixed [98,99]. Metformin significantly decreased urinary F_2_-isoprostanes and increased plasma concentrations of vitamins A and E in Type 2 DM subjects, although no effects were seen in serum malondialdehyde (MDA) and total antioxidant status (TAS) in subjects with polycystic ovarian syndrome after 12-week treatment [49,51]. In the same study, treatment with rosiglitazone was able to increase plasma TAS from 0.95 to 1.21 mmol/l significantly (*p* < 0.005), and decrease plasma MDA from 7.46 to 4.02 nmol/l (*p* < 0.001). In an animal study of Type 2 DM mice, rosiglitazone treatment for 7 days was able to significantly decrease serum F_2_-isoprostanes and vascular superoxide production, and increase vascular catalase concentrations (all *p* < 0.05) [100].

## Type 2 DM, antioxidant status, oxidative stress, and results of antioxidant supplementation trials

There are many reports of decreased antioxidant status in Type 2 DM patients [101–107]. Studies, summarized in Table 5, show also that Type 2 DM is a condition of increased oxidative stress [44,45,103,107–109].

**Table 5.** Human studies showing increased ox stress and/or depleted antioxidant status in Type 2 DM.

Subject characteristics	Biomarkers measured
Type 2 DM subjects and healthy age- and sex- matched controls. Males: Type 2 DM [*n* = 44], control [*n* = 33]; Females: Type 2 DM [*n* = 26], control [*n* = 26] All subjects had normal hepatic and endocrine functions, with HbA1c <6–7%. All patients were taking oral antidiabetic pills during the study period or the past 3–5 years, with no history of other medications. Diabetic duration ∼4.0 years. Exclusion criteria – macro- and microangiopathic complication, coronary heart disease or hypertension, life-threatening diseases such as cancer [110]	Males: Serum MDA significantly higher in DM compared with controls (0.29 vs. 0.11 pmoles/mg protein, *p* < 0.05). Reduced GSH was decreased in the diabetic group (195.6 vs. 294.8 pmoles/mg protein in control, *p* < 0.05) Females: Similar patterns observed in female groups. MDA concentrations were 0.23 vs. 0.07 pmoles/mg protein in controls, *p* < 0.05. Reduced GSH was 193.9 vs. 250.3 pmoles/mg protein in controls, *p* < 0.05.
Type 2 DM patients (HbA1c >7.2%) [*n* = 44] aged 51–65 years, healthy controls [*n* = 12]. 23 patients were previously treated with metformin (16 males, 7 females) and 21 treated with SU (16 males, 5 females) [111]	Plasma MDA and *F*_2_-isoprostanes significantly lower in the metformin treated group compared with SU-treated group, (MDA, 2.82 vs. 3.12 μmol/l, *p* < 0.05; *F*_2_-isoprostanes, 25.2 vs. 31.3 pg/ml, *p* < 0.05). These parameters were significantly lower in the control group compared with diabetic patients (MDA: 2.51μmol/l; *F*_2_-isoprostanes: 17.2 pg/ml in the control group). Plasma TAS was lowered in SU-treated group (1.02μmol/l) compared with metformin group (1.31μmol/l) and control (1.45μmol/l), but was higher in control overall compared with diabetic subjects, *p* < 0.05. SU-treated subjects had the lowest blood total GSH (590μmol/l) which was significantly lower than the metformin group (870μmol/l) and the control (980μmol/l), *p* < 0.05.
Type 2 DM patients’ mean age 52.35 years, mean duration of DM of 6.95 years [*n* = 64], healthy subjects’ mean age 51.02 years [*n* = 36] Exclusion criteria – renal, liver, heart diseases [112]	TBARS concentrations (4.53μmol/l) and SOD activity (4.85U/ml) were elevated in the diabetic group compared with the control group (TBARS: 1.36μmol/l, *p* < 0.001; SOD activity: 0.71, *p* < 0.00001). Plasma catalase activity was significantly decreased in the diabetic group (120k/l) compared with control (152k/l, *p* < 0.00001).
Type 2 DM patients mean age 57.2 years [*n* = 41]. HbA1c ∼9.1% and control subjects with normal glucose tolerance and no family history of DM, [*n* = 33] [113]	The serum 8-OHdG concentrations in DM subjects were significantly higher than in control subjects (5.03 vs. 0.96 pmol/ml, *p* < 0.01). When patients were subdivided into three groups by the presence and severity of retinopathy, subjects with proliferative diabetic retinopathy (8.27pmol/ml) had significantly higher concentrations of 8-OHdG than subjects with NPDR (4.92pmol/ml), or subjects without retinopathy (3.38pmol/ml, *p* < 0.01 vs. no retinopathy and NPDR). Similarly, serum 8-OHG concentrations in patients with azotemia (9.31pmol/ml) were higher than those with normoalbuminuria (3.54pmol/ml), microalbuminuria (5.74pmol/ml), or overt proteinuria (3.40pmol/ml, *p* < 0.01 vs. all).
38 Type 2 DM patients [*n* = 38] and 36 healthy age-matched controls [*n* = 36] Subjects not taking antioxidant supplements [114]	Multiple DNA base oxidation products, but not DNA base de-amination or chlorination products were found to be elevated in white blood cell DNA from DM patients compared with age-matched controls. No significant difference seen in 5-chlorouracil and concentrations of the base de-amination products hypoxanthine and xanthine. 8-hydroxyguanine, 5-OH uracil, 5-OH methyluracil, thymine lycol, 5-OH methyldantoin, 5-OH hydantoin, 5-OH cytosine, 2-OH adenine, 8-OH adenine, and FAPy-adenine concentrations were elevated, whereas FAPy-guanine remained unchanged. Total DNA base oxidative damage products in DM subjects was double than that found in healthy controls, *p* < 0.01.
Type 2 DM [*n* = 24] Age-, sex-, and BMI-matched subjects (obese group) [*n* = 19] Unmatched control group [*n* = 34] Exclusion criteria – presence of Prader–Willi Syndrome, hypothyroidism, known alcohol or drug abuse, congenital CVD, history of malignancy, use of glucocorticoids, and chronic renal failure or known primary renal disease. None of the participants were taking lipid-lowering drugs [115]	Plasma IL-6 concentrations were 29.3% higher in the obese group compared with the control group, DM group had a plasma IL-6 concentration 44.5% higher than the control group and 21.5% higher than the obese group, *p* < 0.001 for all. Ultra-sensitive CRP was not different among the groups. OxLDL was elevated 1.6 fold in the DM group compared with the control (*p* = 0.002). Plasma TAS was not different between the three groups.
Type 2 DM subjects (mean duration of diabetes: 8.5 years HbA1c ∼7.1%) [*n* = 26] 52 age-matched healthy control subjects [*n* = 52] All Type 2 DM patients were free of microangiopathy. Exclusion criteria – smoking, inflammatory diseases, known myocardial infarction, stroke. *8 DM subjects were on with diet and exercise, 18 subjects were on oral hypoglycemic agents [116]	Urinary 8-OHdG (11.1 ng/mg. creatinine) and isoprostane (0.87 ng/g. creatinine) were also significantly higher among diabetic subjects (*p* = 0.019, *p* = 0.009 respectively). Urinary 8OHdG level in control was 8.8 ng/mg. creatinine; urinary isoprostane 0.32 ng.g creatinine.
Type 2 DM patients (33 without diabetic complications and 27 with diabetic retinopathy, of which 21 were classified as non-proliferative diabetic retinopathy, 6 were proliferative diabetic retinopathy) [*n* = 60 in total] Healthy, age-matched control subjects [*n* = 32] Exclusion criteria – acute and chronic infections, fever, malignancy, acute, and chronic nephritis, cirrhosis, and congestive heart failure. All patients were treated with insulin only [117]	Significant higher serum MDA found in DM subjects (6.72 vs 3.12 nmol/ml for controls, *p* < 0.01). Serum CD in DM subjects significantly elevated (0.486 vs. 0.321, *p* < 0.05). DM subjects with retinopathy had higher concentration in MDA and CD compared with DM subjects without complications (7.90 vs. 5.76, *p* < 0.05 for MDA; 0.566 vs. 0.420, *p* < 0.01 for CD). A significantly higher level of 8-OHdG in DM with or without retinopathy (24.96 ng/ml) compared with normal subjects (5.89 ng/ml) was seen. DM subjects with retinopathy had a significantly higher 8-OHdG conc. compared with DM subjects with no vascular complication (26.86 vs. 23.40 ng/ml, *p* < 0.05). Compared with controls, serum AOPP in DM subjects was significantly higher (25.66 vs. 16.66 μmol/l, *p* < 0.01), as was serum protein carbonyl (4.08 vs. 3.12 nmol/ml, *p* < 0.01). DM subjects with retinopathy also had significantly higher AOPP and protein carbonyl compared with DM subjects without vascular complications (27.8 vs. 23.91μmol/l, *p* < 0.05 for AOPP; 4.60 vs. 3.65 nmol/ml, *p* < 0.05 for protein carbonyl).
Type 2 DM subjects (normotensive, treated with only insulin, without diabetic complications, HbA1c ∼7.67%) [*n* = 60] Age-matched healthy controls [*n* = 60]. Exclusion criteria – smoking, oral hypoglycemic agents or other antioxidant therapy [118]	MDA concentrations were significantly increased (∼2-fold) in DM patients compared with normal controls (117.8 vs. 58.9 nM/dl, *p* < 0.001).
Type 2 DM patients (HbA1c ∼12.5%) [*n* = 55] Healthy control subjects [*n* = 40] All patients were treated with oral hypoglycemic agents, and were not taking any other medications or vitamin supplements [119]	TBARS concentrations in DM subjects were significantly higher than in healthy controls (231 vs. 58 μmol/l, *p* < 0.05). Carbonyl content in DM subjects was significantly higher than in controls (12.2 vs. 3.82 nmol/mg pt, *p* < 0.05). Plasma NO was significantly higher in DM subjects when compared to normal subjects (58 vs. 35 μmol/l, *p* < 0.05). The activities of SOD (1340 U/mg), GPx (40U/mg) and CAT (198 nmol H_2_O_2_ oxidized/min/ml) in RBC were significantly lower in T2DM patients compared to normal (1620U/mg, 52U/mg, 266 nmol H_2_O_2_ oxidized/min/ml), *p* < 0.05 respectively). Plasma vitamin A (1.75μmol/l), beta-carotene (2.23μmol/l), Vitamin C (41μmol/l), Vitamin E (16.2μmol/l), and uric acid (0.12 mmol) were all significantly lower in DM patients compared to controls (3.24, 3.86, 78, 21.4 μmol/l, 0.14 mmol/l, *p* < 0.05 for all).

### What is the source of increased oxidative stress in DM?

There is no generally agreed source of the increased oxidative stress found in DM [120]. It has been proposed, but disputed, that acute elevation of plasma glucose is the trigger [120–122]. Others suggest that it is glycemic variability that is the root cause [123,124]. However, conceptually at least, if antioxidant status is high, then oxidative damage to key biomolecules in Type 2 DM might be avoided, improving outcome regardless of the exact relationship between glycemic control and generation of reactive oxygen species. This has led to the suggestion that antioxidant supplementation should be investigated as an adjunct therapy in DM, the hypothesis being that increasing antioxidant defense will lower oxidative stress and help slow or prevent vascular changes that lead to complications [125,126].

### Can antioxidant supplementation decrease oxidative stress in DM?

This has been explored in human supplementation trials with various types and combinations of antioxidants, and using diverse biomarkers of oxidative stress. Some researchers have also investigated the effects of antioxidant supplementation on inflammation, lipids, blood pressure, and glycemic control. These studies (summarized in Table 5) have not revealed clear evidence of benefit. However, none of these studies considered the possible confounding effect of therapy with drugs with antioxidant (or other) effects.

### Evidence of decreased oxidative stress in DM after antioxidant supplementation

In Ward et al*.*’s [127] study, the only oxidative stress biomarker measured was plasma and 24-hour urine F_2_-isoprostanes. It was found that plasma F_2_-isoprostanes were significantly reduced in both the tocopherol treatment groups when compared to the placebo group, but no difference was seen in urinary F_2_-isoprostanes in any of the groups. This could be because urinary F_2_-isoprostane excretion may be confounded by local production in the kidney and is more variable [128].

In another study, vitamin C supplementation had no effect on the vitamin E concentration of HDL-C in patients with diabetes, although an increase in intracellular glutathione (GSH) was seen (*p* < 0.01) when compared to the placebo group [129]. The investigators state that these subjects were on either glibenclamide, metformin, or insulin to control their diabetes, but it is likely that the subjects would also be on a statin or anti-hypertensive drugs [129]. No effect on conjugated dienes, thiobarbituric acid reactive substances (TBARS), or susceptibility of LDL-C or HDL-C to oxidation was seen post supplementation [129]. The increase seen in erythrocyte GSH, a naturally occurring antioxidant, is probably due to the low GSH found in this group before supplementation (0.5 ± 0.7 nmol/mg protein), as the normal range for erythrocyte GSH is between 745 and 1473 μmol/l in healthy individuals aged 12–69 years [130].

Nuttall et al*.*’s study of nine Type 2 DM subjects included the most advanced age cohort and one of two cross-over studies under review here, with 500 mg vitamin C and 400IU vitamin E taken during the first supplementation period and 1000 mg vitamin C and 800IU vitamin E taken during the second period [131]. The study was not placebo controlled [131]. With the exception of plasma antioxidants, lipid peroxides were the only marker of oxidative stress measured [131]. It is noted here that all subjects had advanced complications, including neuropathy, cardiovascular complications, and cerebrovascular disease, and the subjects were taking sulphonylureas, and/or metformin, and insulin, although no subjects were taking lipid-lowering therapy [131]. A bigger decrease (*p* < 0.01) in lipid peroxides was seen after the first (lower dose) supplementation than the second (*p* < 0.05) [131]. The investigators note that the higher doses of vitamins C and E taken during the second supplementation period may have reached the pro-oxidant threshold [131].

Gazis et al*.* [132] supplemented 48 subjects with Type 2 DM and no vascular complications with 1600IU α-tocopherol for 8 weeks. This study was the only one to include individual information on the drugs each subject was using to manage diabetes, but no separate statistical analysis was performed on supplementation outcome taking into consideration the medication history of the subjects. No direct biomarkers of oxidative stress were measured and no significant changes in blood flow or vasodilation were seen. In Tessier et al*.*’s study, 36 subjects with Type 2 DM were randomized into one of three groups, a placebo group, a group taking 0.5 g and a group taking 1 g daily of vitamin C for 12 weeks. DM status was stable in all subjects with HbA1c ≤ 9% [129]. Conjugated dienes, TBARS, vitamin E content of LDL and HDL lipoproteins and granulocyte levels of vitamin C, GSH and glutathione disulfide (GSSG) were measured [129]. After supplementation (both the 0.5 g per day and 1.0 g per day groups), there was a significant increase (*p* < 0.05) in cellular GSH when compared to baseline, although no changes in GSSG were observed [129]. No changes in markers of lipid peroxidation (TBARS and conjugated dienes) were seen [129].

In a supplementation study using mixed tocopherols, 55 Type 2 DM subjects were randomized to take (1) 500 mg α-tocopherol per day, or (2) a combination of 75 mg α-tocopherol, 315 mg γ-tocopherol, and 110 mg of δ-tocopherol, or (3) placebo for a duration of 6 weeks [133]. Plasma and urinary F_2_-isoprostanes and erythrocyte SOD and glutathione peroxidase (GPx) were measured at baseline and at the completion of the supplementation period [133]. Treatment with either α-tocopherol or the mixed tocopherols significantly decreased plasma F_2_-isoprostanes when compared with the placebo group, (*p* < 0.001), although neither treatment affected urinary F_2_-isoprostanes [133]. There were no changes in SOD and GPx post supplementation of either α-tocopherol or mixed tocopherols [133]. Diabetes status in this population was well controlled, with mean HbA1c at around 6.6% [133]. Although the investigators documented the percentage of subjects taking oral hypoglycemics, anti-hypertensives, lipid-lowering drugs, aspirin, and statins, analysis of the data did not take account any influence these drugs may have had on the results. In a study by Ble-Castillo et al., Type 2 DM subjects were randomized to receive 800IU α-tocopherol per day (*n* = 13) or placebo (*n* = 21) for 6 weeks [134]. Subjects on insulin, hormonal therapy, antioxidant supplements, smokers, and with hypertensive were excluded [134]. The investigators reported a 46% reduction in erythrocyte MDA and a significant increase (*p* < 0.001) in serum total antioxidant in the α-tocopherol group post supplementation when compared to baseline [134]. These results are in conflict with other studies investigating the effect of antioxidant supplementation on oxidative stress markers in subjects with Type 2 DM and may be unreliable. The actual concentrations of serum TAS and the erythrocyte MDA were not given, and the results are simply shown in graphical form. In addition, the serum-TAS concentrations reported are lower than the range given by the manufacturers of the kit (Randox) by a factor of a thousand.

In summary, vitamin C and vitamin E are the most popular antioxidants used in supplementation studies in subjects with Type 2 DM. The supplementation period of studies reviewed here is between 3 and 12 weeks. Ascorbic acid depletion–repletion studies have shown that both plasma and intracellular ascorbic acid will reach saturation at doses of 500 mg per day within about 40 days of supplementation, which is around 6 weeks [135]. This could mean plasma and tissue concentrations of ascorbic acid would not have reached optimal levels for that dose in studies with a supplementation period of less than 6 weeks, although the doses used are many fold higher than the recommended daily amount (RDA) of up to 90 mg [136]. All studies using vitamin E also supplemented subjects with much more than the RDA of 22.5IU (15 mg) [137].

Although all studies reviewed in this section (and summarized in Table 6) are antioxidant supplementation studies, the study population are diverse. Ages ranged from 40 to 77 years, and while some used subjects who were comparatively complication-free, subjects used in other studies had suffered serious micro- and macro-vascular complications [6,131].

**Table 6.** Antioxidant supplementation studies in Type 2 DM subjects.

Subjects characteristics/ test material	Study description	Outcome/biomarkers investigated	Information on existing treatment
Type 2 DM subjects meeting the following criteria: DM onset after 35 years of age, at least 5-year disease without insulin use, FPG ≧7.8 mmol/l, BMI <33 kg/m^2^, serum creatinine <2.0 mg/dl, Exclusion criteria included history of ketoacidosis/onset of diabetes before age 35, smoking, use of vitamin supplements, treatment with glucocorticoids, thyroid hormone, lipid-lowering agents, or other drugs known to affect serum lipids, urine protein excretion >250 mg/d and serum α-tocopherol levels >28μmol/l [138]	Subjects received placebo, 750 mg sucrose per capsule as washout, then antioxidant treatment, (24 mg β-carotene, 1000 mg ascorbic acid, and 800IU α-tocopherol/day) Subjects followed post supplementation for 8 weeks. Treatment duration, placebo for 8 weeks and antioxidant for 12 weeks. Normal control subjects, age- and sex-matched to DM subjects [*n* = 20]	Antioxidant treatment had no effect on glycemic control, glycohemoglobin or serum lipids, but significantly increased LDL oxidation lag phase from 55 to 129 min; conjugated dienes decreased from 1.23 to 0.62 OD units; TBARS decreased from 78 to 33 nmol MDA/mg LDL protein (*p* < 0.0001 compared to control group).	No information given
Type 2 DM subjects and healthy controls Exclusion criteria included smoking, use of antioxidant supplements, hypolipidemic drugs, beta-blockers, or NSAID, renal or liver dysfunction, and alcohol consumption > 1oz/day [139]	All subjects supplemented with RRR-α -tocopherol (1200IU/day), following a 2-month washout. (1) Type 2 DM subjects without macrovascular complications [*n* = 24] (2) Type 2 DM patients with macrovascular complications [*n* = 23] (3) Age-, sex-, and BMI- matched healthy control subjects [*n* = 25] Treatment duration, 3 months.	hsCRP decreased in all three groups after supplementation compared to both baseline and washout phases (*p* < 0.02). IL-6 concentrations significantly decreased following supplementation in all three groups, *p* < 0.02. Compared to matched controls, group 2 showed significantly elevated hsCRP, all DM patients had increased levels of monocyte IL-6.	No information given
Type 2 DM subjects with urinary albumin excretion rate between 30 and 300 mg/24 h. Exclusion criteria included SBP > 200mmHg, DBP > 110mmHg, GFR < 40 ml/min/1.73 m^2^ , prior MI or congestive heart failure. Treatment with ACE-inhibitors (*n* = 28)/ vitamin E and C supplements (*n* = 3) was stopped and BP was strictly monitored and controlled at < 180 mmHg at least 8 weeks before study commenced [6]	(1) Treatment group (total dose of 1250 mg vitamin C and 680 IU of d-α-tocopherol/day) [*n* = 15] (2) Placebo group, 5 matching placebo tablets [*n* = 14]	A significant decrease in urinary albumin excretion rate from 243 in the placebo to 197 mg/24 h, (*p* = 0.04) in the treatment group. No significant changes were seen in blood pressure, HbA1c, FPG, serum creatinine, and cholesterol with active treatment.	No information given
Type 2 DM subjects (FPG ≥ 7.8 mmol/l, a mean of 4.5 years for diabetes) Exclusion criteria –included evidence of cerebrovascular, peripheral vascular or ischemic heart disease, diabetic retinopathy requiring treatment, BP > 160/90mmHg, TC > 7.5 mmol/l, urinary albumin:creatinine ratio elevated (> 2.5 mg/mmol creatinine for men, > 3.5 for women) or serum creatinine > 100μmol/l [132]	Subjects randomized to (1) 1600IU α-tocopherol, [*n* = 48] (2) Placebo, [*n* = 21] Treatment duration, 8 weeks.	α-tocopherol treatment increased plasma vitamin E from 28.2 to 65.9 μmol/l (*p* < 0.01). No significant changes in basal blood flow or on vasodilation to low or high doses of the endothelium-independent vasodilator sodium nitroprusside or the endothelium-dependent vasodilators acetylcholine and bradykinin, were seen in either group.	48 subjects were either treated with diet alone [*n* = 17], sulphonylureas [*n* = 20], metformin [*n* = 5], or both [*n* = 6].
Patients with uncomplicated Type 2 DM controlled by diet or oral hypoglycemics Exclusion criteria, SBP > 160mmHg, DBP >90 mmHg, TC > 7.0 mmol/l, proteinurea, significantly impaired renal function, clinical evidence of neuropathy or atherosclerosis [140]	Subjects randomized to (1) Vitamin C (1500 mg/day) [*n* = 18] (2) Placebo [*n* = 17] Treatment duration, 3 weeks	Plasma ascorbic acid increased from 58 at baseline to 122 μmol/l, *p* < 0.01 following supplementation. Plasma 8-epi-PGF2α, blood pressure, lipid profiles, and endothelial function were not significantly different from baseline/placebo following vitamin C supplementation.	Metformin, sulphonylureas, ACE inhibitors, diuretics, calcium channel blockers
Type 2 DM patients with diabetic complications, retinopathy (7), neuropathy (5), nephropathy (2) and cataract (1). All subjects were with evidence of cardiovascular complications: hypertension (8), atrial fibrillation (2), angina (5), heart failure (1), cerebrovascular disease (4) and PVD (3) and one with below knee amputation [131]	Cross-over study [*n* = 9] first treatment – 500 mg vitamin C and 400IU vitamin E/day Treatment duration, 4 weeks; 4-week washout period. Second treatment – 1000 mg vitamin C and 800IU vitamin E/day Treatment duration, 4 weeks.	Vitamin E level increased after moderate dose and further after higher dose, 59.8 vs 36.4µmol/l, *p* < 0.001 and 72.7 vs 30.8µmol/l, *p* < 0.001, respectively. Total antioxidant capacity increased after treatment 508.2 vs 436.4 µmol/l trolox eq., *p* < 0.01 (moderate); 519.3 vs 440.8 µmol/l trolox eq., *p* < 0.01 (high). Lipid hydroperoxides were decreased more after the 1st, than after the 2nd treatment (6.1 vs. 12.1 μmol/l, *p* < 0.01; 8.0 vs. 11.6 μmol/l, *p* < 0.05).	Individual medication details are not given, but subjects were on sulphonylureas and/or metformin or insulin, or diet alone.
Female Type 2 DM patients. Exclusion criteria included insulin treatment, hypertension, hormonal therapy, smoking and ingestion of antioxidant supplements at least 3 months before the study, ingestion of hypolipidemic, and NSAID, intake of garlic or fish oil, renal or liver dysfunction poorly accessible veins and alcohol consumption [134]	(1) 800IU α -tocopherol/day [*n* = 13] (2) Placebo [*n* = 21] Treatment duration, 6 weeks	α -tocopherol did not significantly alter concentrations urea, creatinine, uric acid, HDL, serum MDA and HbA1c. A 46% reduction in erythrocyte MDA was seen in the treatment group in comparison with only 10% in placebo, *p* < 0.0001. A reduction in erythrocyte CuZn-SOD activity (from 1083 to 876.4 U/g, *p* < 0.0001) seen in the treatment group	All subjects were treated with oral hypoglycemics or diet, but no information was available as to what treatment each subject was on
Subjects were previously diagnosed of Type 2 DM or were on oral hypoglycemic medication. Exclusion criteria included prior or current use of vitamin E/tocopherol supplements, Type 1 DM, use of insulin, previous coronary or cerebrovascular event ≦6 months, smoking, premenopausal women regular use of nitrate medication, anti-inflammatories, BMI ≧ 35 kg/m^2^, elevated serum creatinine or alcohol intake (> 40 g/day men or > 30 g/day for women) [127]	Randomized in a double-blind, placebo-controlled trial to (1) 500 mg RRR-α-tocopherol/day [*n* = 19] (2) 500 mg mixed tocopherols (60% γ-, 25% δ- and 15% α-tocopherol)/day [*n* = 20] (3) Placebo [*n* = 19] Treatment duration, 6 weeks *3-week washout period prior to the study. Subjects ceased all vitamin, antioxidant/ fish oil supplements	Treatment with α-tocopherol and mixed tocopherols significantly increased blood pressure and heart rate versus placebo. There was no significant difference between the two treatment groups. Plasma total F_2_-isoprostane significantly reduced following either treatment versus placebo, but urinary F_2_-isoprostane not affected.	Antihypertensives, oral hypoglycemics, aspirin, lipid lowering therapy, BP treatment
Type 2 DM patients Exclusion criteria included body mass index (in kg/m^2^) > 35; use of insulin, nonsteroidal anti-inflammatory drugs, or nitrate medication; smoking; use of vitamin E supplements; any recent (in previous 6mo) coronary or cerebrovascular event; impaired renal function (serum creatinine > 110μmol/l for men and > 100μmol/l for women); and alcohol intake > 40 g/d for men and > 30 g/d for women. All volunteers ceased consuming any vitamin, antioxidant, or fish oil supplements for ≥ 3wk before study entry [133]	Subjects randomized to (1) 500 mg RRR-α-tocopherol/day [*n* = 18] (2) 500 mg mixed tocopherols (75 mg α-, 315 mg γ- and 110 mg δ- tocopherol) [*n* = 19] (3) Placebo [*n* = 18] Treatment duration, 6 weeks *3-week washout period prior to the study. Subjects ceased all vitamin, antioxidant/ fish oil supplements	Plasma total F_2_-isoprostane significantly (*P* = 0.001) reduced following either treatment versus placebo, but urinary F_2_-isoprostane not affected. No significant changes seen in erythrocyte SOD and GPx after any supplementation regimen.	Percentage of participants on oral hypoglycemics, (58%), antihypertensives, (51%), lipid-lowering drugs, (53%), aspirin, (38%).
Ambulatory outpatients > 65 years of age with no acute cardiovascular, neurologic event in the prior 6 months, no history of active tobacco smoking and no oral intake of vitamin supplements in the last 4 weeks. Medical treatment of these subjects was stable for the last 3 months with an HbA1c ≦ 9% [*n* = 36] [129]	Subjects randomized to (1) Placebo [*n* = 12] (2) Vitamin C (0.5 g/day) [*n* = 12] (3) Vitamin C (1 g/day) [*n* = 12] Treatment duration, 12 weeks	Significant increase in cellular GSH was observed (0.60 vs. 0.33 nmol/mg protein in the placebo group, *p* < 0.01) in patients on 0.5 g vitamin C/day. Significant increases in cellular GSH (0.93 vs. 0.33 nmol/mg proteins in the placebo, *p* < 0.01), seen in those on 1.0 g vitamin C/day. Vitamin C had no effect on the vitamin E content of HDL in any group. At week 12, no significant difference was seen in lipid peroxidation/oxidative stress markers (conjugated dienes, TBARS) and susceptibility of LDL and HDL to oxidation in the two treatment groups compared to placebo.	Individual medication details are not given, but subjects on glibenclamide, metformin or insulin
Type 2 DM patients with diabetes for >= 1 year and no clinical evidence of overt vascular diseases, acute or chronic inflammatory diseases [141]	Subjects randomized to (1) Vitamin C (1 g/day) [*n* = 14] (2) Acetate cellulose placebo 1000 mg/day, [*n* = 13] Treatment period, 6 weeks	MDA significantly decreased at fasting (*p* = 0.006) and postprandial states (*p* < 0.001) in vitamin C group, compared to placebo group. No significant differences were seen in fasting and postprandial lipid profile.	Subjects were on standard oral hypoglycemic agents, none on lipid-lowering drugs, hormone replacement therapy and diuretics, β-blockers, and aspirin.
Type 2 DM patients with no history of other chronic diseases, kidney stones, hyperparathyroidism, pregnancy and lactation, current insulin treatment and treatment for weight reduction [142]	(1) Vitamin E (800 mg/day) [*n* = 37] (2) Placebo[*n* = 34] Treatment duration, 2 months	Tg decreased significantly in vitamin E group (212 ± 85 mg/dl), compared to placebo group (254 ± 170 mg/dl). No significant differences were seen in serum fasting glucose, HbA1c and β-cell function after vitamin E supplementation, compared to placebo group.	No information given

With all these differences in age, methods of DM management and inclusion/exclusion criteria in the study populations and outcomes assessed to determine benefit, it is very difficult to compare results across studies. Most investigators excluded subjects who were already taking the supplement in question, while some asked subjects to stop taking any type of antioxidant supplements weeks prior to the start of the study [129,134]. Gaede et al*.* [6] asked their subjects to stop taking vitamin C and E and ACE-inhibitors 8 weeks prior to the start of the supplementation period, although hypoglycemic drugs continued to be used. However, Levine et al. did not specify such exclusion criteria [136]. It is noted here that the subjects with Type 2 DM in Darko et al*.*’s study were not deficient in vitamin C, with mean plasma concentrations of 58 ± 6 µmol/l, which is higher than the normal range of 23–50 µmoll [140,143]. The fact that the subjects were not deficient in vitamin C may have contributed to the lack of benefit seen in the only biomarker of oxidative stress measured, plasma F_2_-isoprostanes.

Although some studies collected information on the polypharmacy used in the management of Type 2 DM in the subjects studied, none of the investigators analyzes data taking into account the medication of the subjects.

With the exception of one study, it is clear that supplementing Type 2 DM subjects with oral antioxidants will not further decrease oxidative stress, as seen by the oxidative stress markers measured. It is noted though that none of the above studies looked at subjects who were on oral hypoglycemics, insulin, or statins separately from those subjects who were not taking any of these medications.

In the only antioxidant supplementation study involving patients with Type 1 DM, Beckman et al*.* gave, vitamin C (1 g) and vitamin E (800IU), per day, or placebo to Type 1 DM subjects (*n* = 26), Type 2 DM subjects (*n* = 23), and healthy matched controls (*n* = 45) for 6 months [144]. In those with Type 1 DM, antioxidant supplementation increased endothelium-dependent vasodilation (*p* = 0.023) when compared to baseline, whereas no such effect was seen in those with Type 2 DM [144]. Although no mention was given as to the number of Type 2 DM subjects who were on oral hypoglycemics, it is surmised that the majority of them would be on polypharmacy, whereas the Type 1 DM subjects would most likely be solely on injected insulin therapy. A search of the literature did not result in any studies which have investigated any antioxidant effect of insulin. Beckman et al*.* did note, however, that at baseline, the Type 1 DM subjects were younger, had lower total cholesterol (TC), glucose, and mean arterial pressure than the Type 2 DM subjects [144].

It cannot be discounted, therefore, that differences in age, cholesterol, and glucose concentrations contributed to the difference seen in the result, but it is proposed here that it is possible that the Type 2 DM subjects who are likely to be on oral hypoglycemics and statins would have reached antioxidant-effect-saturation. This does not mean that their tissues are saturated with antioxidants, but it might be feasible that, even if the tissue antioxidant concentration was increased, no further benefits will be seen. This is shown clearly in two controlled studies, De Caterina et al. [59] and Pereira et al. [61], summarized in Table 4, where no further oxidative stress-lowering effects were seen when vitamin E was added to the regimen of subjects taking statins.

It should be taken into consideration also that in health, the balance between reactive species and antioxidant defenses lies slightly in favor of the reactive species so that they are able to fulfill their biological roles and artificially increasing the antioxidant concentration may possibly result in deleterious effects [145].

## Drugs used in the management of Type 2 diabetes and their mechanisms of antioxidant action

Sulfonylureas stimulate insulin release by binding to the sulfonylurea receptor, a subunit of the ATP-dependent K^+^ (K_ATP_) channel complex on the cell membrane of pancreatic β-cells, causing an increase in intracellular calcium, which in turn increases the secretion of pro-insulin. Gliclazide is known to be a free-radical scavenger, and an *in vivo* study of 44 Type 2 DM subjects taking gliclazide for 10 months resulted in a decrease in 8-isoprostanes, a marker of lipid oxidation, and an increase in the total antioxidant capacity and SOD [146,147]. Other sulfonylureas, including glipizide tolazamide, and glibenclamide do not exhibit antioxidant activity [148].

Thiazolidinediones (TZD) work to decrease insulin resistance by binding to peroxisome proliferator-activated receptors gamma (PPARγ). These receptor molecules are found inside the cell nucleus, and when activated migrate to DNA and activate the transcription of a number of genes, including SOD and catalase [149–151]. In addition to being able to indirectly upregulate antioxidant genes, certain TZD, for example, troglitazone, may be able to exert direct antioxidant effects through a side chain which resembles α-tocopherol [152,153]. All TZDs exhibit intracellular antioxidant properties as they are able to reduce NO production through the transrepression of iNOS [153,154].

Biguanides decrease hepatic glucose output and increase the uptake of glucose by skeletal muscle. Metformin exerts its antioxidant effect through mechanisms other than direct radical scavenging [155]. *In vitro* experiments have shown that metformin is able to prevent the formation of advanced glycation end-products when albumin was incubated in the presence of dicarbonyl compounds [156]. *In vitro* experiments with endothelial cells grown in hyperglycemic (10 mmol) conditions have shown that co-incubating the cells with metformin (20 mmol) can inhibit the formation of NAD(P)H oxidase, and therefore, decrease production of hydrogen peroxide [157]. Co-incubation with metformin also leads to an increase in the activity of catalase in both euglycemic and hyperglycemic conditions [157].

Alpha-glucosidase inhibitors slow the digestion of starch in the small intestine so that glucose can enter the bloodstream more slowly. Alpha-glucosidase inhibitors in use include acarbose and miglitol. To date, no antioxidant effects have been reported in these drugs.

Meglitinides, including repaglinide and nateglinide, bind to a K_ATP_ channel on the cell membrane of pancreatic β-cells in a similar manner to sulfonylureas but at a separate binding site. Meglitinides have not been shown to possess any antioxidant properties.

Statins act by competitively inhibiting HMG-CoA reductase and decreasing cholesterol synthesis in the liver. By inhibiting Rac isoprenylation, statins can lead to a reduction in NAD(P)H oxidase and generation of reactive oxygen species [158,159]. In an animal study, simvastatin was able to decrease the formation of superoxide by inhibiting Rac1, a signaling protein involved in cell growth, cell cycle, cell–cell adhesion, and the activation of protein kinases [160].

## Pleiotropic effects of diabetic polypharmacy and their possible confounding effects on observational and supplementation trials of antioxidants

*In vitro*, animal and human studies have been conducted on the pleiotropic, and in particular, the antioxidant effects of statins and hypoglycemic drugs. Tables 1 and 2 summarize the data on statins and hypoglycemic drugs, concentrating on studies which have looked at changes in antioxidant/oxidative stress balance. From the controlled studies of statin ingestion with antioxidant vitamins, some insight into whether these pharmaceuticals are co-operators, confounders, or concealers of oral antioxidants taken in supplement form is offered.

## Conclusions

Evidence that Type 2 DM subjects are in a state of oxidative stress is unequivocal. Short-term supplementation with antioxidant vitamins C and/or E has not further decreased oxidative stress markers in Type 2 DM subjects. That is not to say that antioxidant vitamin supplementation has no role in Type 2 DM, but the effects of these antioxidants may be concealed or confounded by the cocktail of medications already being taken by the patient, and pharmacological agents themselves may have strong antioxidant effects. Further study is required to ascertain if oral antioxidant vitamin supplementation can be a cheap and safe method which can benefit those Type 2 DM subjects whose diabetes, hypertension, and hypercholesterolemia is being controlled by diet alone.

## References

[1] Chen L, Magliano DJ, Zimmet PZ. The worldwide epidemiology of type 2 diabetes mellitus – present and future perspectives. Nat Rev Endocrinol. 2012;8(4):228–336. 10.1038/nrendo.2011.183 22064493

[2] Skrha J. Diabetes mellitus – a global pandemic. Keynote lecture presented at: the Wonca conference; 2013 June; Prague. Eur J Gen Pract. 2014;20(1):65–68. 24351018 10.3109/13814788.2013.847083

[3] van Dieren S, Beulens JW, van der Schouw YT, et al. The global burden of diabetes and its complications: an emerging pandemic. Eur J Cardiovasc Prev Rehanil. 2010;17(Suppl 1):S3–S8. 10.1097/01.hjr.0000368191.86614.5a 20489418

[4] Ramachandran A, Snehalatha C, Shetty AS, et al. Trends in prevalence of diabetes in Asian countries. World J Diabetes. 2012;3(6):110–117. 22737281 10.4239/wjd.v3.i6.110 PMC3382707

[5] Kerner W, Bruckel J. German diabetes A. Definition, classification and diagnosis of diabetes mellitus. Exp Clin Endocrinol Diabetes. 2014;122(7):384–386. 25014088 10.1055/s-0034-1366278

[6] Gaede P, Lund-Andersen H, Parving HH, et al. Effect of a multifactorial intervention on mortality in type 2 diabetes. N Engl J Med. 2008;358(6):580–591. 18256393 10.1056/NEJMoa0706245

[7] Blum A, Shamburek R. The pleiotropic effects of statins on endothelial function, vascular inflammation, immunomodulation and thrombogenesis. Atherosclerosis. 2009;203(2):325–330. 18834985 10.1016/j.atherosclerosis.2008.08.022

[8] Davignon J, Jacob RF, Mason RP. The antioxidant effects of statins. Coronary Artery Dis. 2004;15(5):251–258. 10.1097/01.mca.0000131573.31966.34 15238821

[9] Elisaf M. Effects of fibrates on serum metabolic parameters. Curr Med Res Opin. 2002;18(5):269–276. 12240789 10.1185/030079902125000516

[10] Franke SI, Muller LL, Santos MC, et al. Vitamin C intake reduces the cytotoxicity associated with hyperglycemia in prediabetes and type 2 diabetes. BioMed Res Int. 2013;2013:896536. 23984417 10.1155/2013/896536 PMC3741954

[11] Lam CS, Benzie IF, Choi SW, et al. Relationships among diabetic retinopathy, antioxidants, and glycemic control. Optom Vis Sci. 2011;88(2):251–256. 21217409 10.1097/OPX.0b013e318208494a

[12] Prentki M, Nolan CJ. Islet β cell failure in type 2 diabetes. J Clin Invest. 2006;116(7):1802–1812. 16823478 10.1172/JCI29103 PMC1483155

[13] American Diabetes A Diagnosis and classification of diabetes mellitus. Diabetes Care. 2010;33(Suppl1):62–69.

[14] Shin D, Cho YM, Lee S, et al. Pharmacokinetic and pharmacodynamic interaction between gemigliptin and metformin in healthy subjects. Clin Drug Investig. 2014;34(6):383–393. 10.1007/s40261-014-0184-3 24627290

[15] Dhawan S, Singh B, Garg SK, et al. Bioavailability of immediate- and extended-release formulations of glipizide in healthy male volunteers. Clin Pharmacokinet. 2006;45(3):317–324. 16509763 10.2165/00003088-200645030-00007

[16] Huber M, Bolbrinker J, Kreutz R. Pharmacokinetics and safety of olmesartan medoxomil in combination with glibenclamide in healthy volunteers. Clin Exp Hypertens. 2006;28(7):631–633. 17060062 10.1080/10641960600946171

[17] Gross AS, Bridge S, Shenfield GM. Pharmacokinetics of tolbutamide in ethnic Chinese. Br J Clin Pharmacol. 1999;47(2):151–156. 10190649 10.1046/j.1365-2125.1999.00868.x PMC2014169

[18] Karara AH, Dunning BE, McLeod JF. The effect of food on the oral bioavailability and the pharmacodynamic actions of the insulinotropic agent nateglinide in healthy subjects. J Clin Pharmacol. 1999;39(2):172–179. 11563410 10.1177/00912709922007606

[19] Hatorp V, Oliver S, Su CA. Bioavailability of repaglinide, a novel antidiabetic agent, administered orally in tablet or solution form or intravenously in healthy male volunteers. Int J Clin Pharmacol Ther. 1998;36(12):636–641. 9877000

[20] Chu KM, Hu OY, Pao LH, et al. Pharmacokinetics of oral rosiglitazone in Taiwanese and post hoc comparisons with Caucasian, Japanese, Korean, and mainland Chinese subjects. J Pharm Pharm Sci. 2007;10(4):411–419. 18261363 10.18433/j3159d

[21] Jaakkola T, Backman JT, Neuvonen M, et al. Effect of rifampicin on the pharmacokinetics of pioglitazone. Br J Clin Pharmacol. 2006;61(1):70–78. 16390353 10.1111/j.1365-2125.2005.02515.x PMC1884976

[22] Xu P XD, Zhu Y, Wang F, et al. Pharmacokinetic properties and bioequiavailability of miglitol in Chinese subjects. Chinese J Hosp Pharm. 2005;25(5):409–411.

[23] Krishna R, Bergman A, Larson P, et al. Effect of a single cyclosporine dose on the single-dose pharmacokinetics of sitagliptin (MK-0431), a dipeptidyl peptidase-4 inhibitor, in healthy male subjects. J Clin Pharmacol. 2007;47(2):165–174. 17244767 10.1177/0091270006296523

[24] Lin Y, Sun Z. Current views on type 2 diabetes. J Endocrinol. 2010;204(1):1–11. 19770178 10.1677/JOE-09-0260 PMC2814170

[25] Mignini F, Tomassoni D, Streccioni V, et al. Pharmacokinetics and bioequivalence study of two tablet formulations of lovastatin in healthy volunteers. Clin Exp. 2008;30(2):95–108. 10.1080/10641960801944249 18293165

[26] Deng S, Chen XP, Cao D, et al. Effects of a concomitant single oral dose of rifampicin on the pharmacokinetics of pravastatin in a two-phase, randomized, single-blind, placebo-controlled, crossover study in healthy Chinese male subjects. Clin Ther. 2009;31(6):1256–1263. 19695392 10.1016/j.clinthera.2009.06.006

[27] Teng R, Mitchell PD, Butler KA. Pharmacokinetic interaction studies of co-administration of ticagrelor and atorvastatin or simvastatin in healthy volunteers. Eur J Clin Pharmacol. 2013;69(3):477–487. 22922682 10.1007/s00228-012-1369-4

[28] Spence JD, Munoz CE, Hendricks L, et al. Pharmacokinetics of the combination of fluvastatin and gemfibrozil. Am J Cardiol. 1995;76(2):80–3A. 7604806 10.1016/s0002-9149(05)80024-4

[29] Bergman E, Matsson EM, Hedeland M, et al. Effect of a single gemfibrozil dose on the pharmacokinetics of rosuvastatin in bile and plasma in healthy volunteers. J Clin Pharmacol. 2010;50(9):1039–1049. 20150523 10.1177/0091270009357432

[30] Martin PD, Dane AL, Schneck DW, et al. An open-label, randomized, three-way crossover trial of the effects of coadministration of rosuvastatin and fenofibrate on the pharmacokinetic properties of rosuvastatin and fenofibric acid in healthy male volunteers. Clin Ther. 2003;25(2):459–471. 12749507 10.1016/s0149-2918(03)80089-9

[31] Kosoglou T, Zhu Y, Statkevich P, et al. Assessment of potential pharmacokinetic interactions of ezetimibe/simvastatin and extended-release niacin tablets in healthy subjects. Eur J Clin Pharmacol. 2011;67(5):483–492. 21120461 10.1007/s00228-010-0955-6

[32] Heintz B, Verho M, Brockmeier D, et al. Multiple-dose pharmacokinetics of ramipril in patients with chronic congestive heart failure. J Cardiovasc Pharmacol. 1993;22(Suppl 9):S36–S42. 7514239

[33] Tamimi JJ S, Alam SM II, et al. Bioequivalence evaluation of two brands of lisinopril tablets (Lisotec and Zestril) in healthy human volunteers. Biopharm Drug Dispos. 2005;26(8):335–339. 16075412 10.1002/bdd.465

[34] Rohatagi S, Lee J, Shenouda M, et al. Pharmacokinetics of amlodipine and olmesartan after administration of amlodipine besylate and olmesartan medoxomil in separate dosage forms and as a fixed-dose combination. J Clin Pharmacol. 2008;48(11):1309–1322. 18974285 10.1177/0091270008322176

[35] Riess W, Dubach UC, Burckhardt D, et al. Pharmacokinetic studies with chlorthalidone (Hygroton) in man. Eur J Clin Pharmacol. 1977;12(5):375–382. 598410 10.1007/BF00562454

[36] Miao Y, Ottenbros SA, Laverman GD, et al. Effect of a reduction in uric acid on renal outcomes during losartan treatment: a post hoc analysis of the reduction of endpoints in non-insulin-dependent diabetes mellitus with the angiotensin II antagonist losartan trial. Hypertension. 2011;58(1):2–7. 21632472 10.1161/HYPERTENSIONAHA.111.171488

[37] Bain SC, Le Guen CA, Lunec J, et al. Comparison of the free radical scavenging activity of captopril versus enalapril: a three month in vivo study in hypertensive diabetic patients. J Hum Hypertens. 1991;5(6):511–515. 1791611

[38] Abou-Auda HS. Comparative pharmacokinetics and pharmacodynamics of furosemide in Middle Eastern and in Asian subjects. Int J Clin Pharmacol Ther. 1998;36(5):275–281. 9629992

[39] Xu T, Bao S, Geng P, et al. Determination of metoprolol and its two metabolites in human plasma and urine by high performance liquid chromatography with fluorescence detection and its application in pharmacokinetics. J Chronomatogr B Analyt Technol Biomed Life Sci. 2013;937:606. 10.1016/j.jchromb.2013.08.017 24018320

[40] Cainazzo MM, Pinetti D, Savino G, et al. Pharmacokinetics of a new extended-release nifedipine formulation following a single oral dose, in human volunteers. Drugs Exp Clin Res. 2005;31(3):115–121. 16033250

[41] Lilja JJ, Juntti-Patinen L, Neuvonen PJ. Effect of rifampicin on the pharmacokinetics of atenolol. Basic Clin Pharmacol Toxicol. 2006;98(6):555–558. 16700816 10.1111/j.1742-7843.2006.pto_379.x

[42] Benzie IF, Strain JJ. The ferric reducing ability of plasma (FRAP) as a measure of “antioxidant power”: the FRAP assay. Anal Biochem. 1996;239(1):70–76. 8660627 10.1006/abio.1996.0292

[43] Benzie IF, Choi SW. Antioxidants in food: content, measurement, significance, action, cautions, caveats, and research needs. Adv Food Nutr Res. 2014;71:1–53. 24484938 10.1016/B978-0-12-800270-4.00001-8

[44] Al-Aubaidy HA, Jelinek HF. Oxidative DNA damage and obesity in type 2 diabetes mellitus. Eur J Endocrinol. 2011;164(6):899–904. 21436346 10.1530/EJE-11-0053

[45] Butkowski EG, Al-Aubaidy HA, Jelinek HF. Interaction of homocysteine, glutathione and 8-hydroxy-2'-deoxyguanosine in metabolic syndrome progression. Clin Biochem. 2017;50(3):116–120. 27751791 10.1016/j.clinbiochem.2016.10.006

[46] Liu XH, Li XM, Han CC, et al. Effects of combined therapy with glipizide and Aralia root bark extract on glycemic control and lipid profiles in patients with type 2 diabetes mellitus. J Sci Food Agr. 2015;95(4):739–744. 25042995 10.1002/jsfa.6829

[47] Fava D, Cassone-Faldetta M, Laurenti O, et al. Gliclazide improves anti-oxidant status and nitric oxide-mediated vasodilation in Type 2 diabetes. Diabet Med. 2002;19(9):752–757. 12207812 10.1046/j.1464-5491.2002.00762.x

[48] Signorini AM, Fondelli C, Renzoni E, et al. Antioxidant effects of gliclazide, glibenclamide, and metformin in patients with type 2 diabetes mellitus. Curr Ther Res Clin E. 2002;63(7):411–420.

[49] Formoso G, De Filippis EA, Michetti N, et al. Decreased in vivo oxidative stress and decreased platelet activation following metformin treatment in newly diagnosed type 2 diabetic subjects. Diabetes Metab Res Rev. 2008;24(3):231–237. 17966969 10.1002/dmrr.794

[50] Memisogullari R, Turkeli M, Bakan E, et al. Effect of metformin or gliclazide on lipid peroxidation and antioxidant levels in patients with diabetes mellitus. Turk J Med Sci. 2008;38(6):545–548.

[51] Yilmaz M, Bukan N, Ayvaz G, et al. The effects of rosiglitazone and metformin on oxidative stress and homocysteine levels in lean patients with polycystic ovary syndrome. Hum Reprod. 2005;20(12):3333–3340. 16123091 10.1093/humrep/dei258

[52] Manning PJ, Sutherland WH, Walker RJ, et al. The effect of rosiglitazone on oxidative stress and insulin resistance in overweight individuals. Diabetes Res Clin Pract. 2008;81(2):209–215. 18541328 10.1016/j.diabres.2008.04.015

[53] Tankova T, Koev D, Dakovska L, et al. The effect of repaglinide on insulin secretion and oxidative stress in type 2 diabetic patients. Diabetes Res Clin Pract. 2003;59(1):43–49. 12482641 10.1016/s0168-8227(02)00179-1

[54] Mirmiranpour H, Mousavizadeh M, Noshad S, et al. Comparative effects of pioglitazone and metformin on oxidative stress markers in newly diagnosed type 2 diabetes patients: a randomized clinical trial. J Diab Compl. 2013;27(5):501–507. 10.1016/j.jdiacomp.2013.05.006 23891275

[55] Skrha J, Prazny M, Hilgertova J, et al. Oxidative stress and endothelium influenced by metformin in type 2 diabetes mellitus. Eur J Clin Pharmacol. 2007;63(12):1107–1114. 17874238 10.1007/s00228-007-0378-1

[56] Chakraborty A, Chowdhury S, Bhattacharyya M. Effect of metformin on oxidative stress, nitrosative stress and inflammatory biomarkers in type 2 diabetes patients. Diabetes Res Clin Pract. 2011;93(1):56–62. 21146883 10.1016/j.diabres.2010.11.030

[57] Nakhjavani M, Morteza A, Asgarani F, et al. Metformin restores the correlation between serum-oxidized LDL and leptin levels in type 2 diabetic patients. Redox Rep. 2011;16(5):193–200. 22005339 10.1179/1351000211Y.0000000008 PMC6837745

[58] Esteghamati A, Eskandari D, Mirmiranpour H, et al. Effects of metformin on markers of oxidative stress and antioxidant reserve in patients with newly diagnosed type 2 diabetes: a randomized clinical trial. Clin Nutr. 2013;32(2):179–185. 22963881 10.1016/j.clnu.2012.08.006

[59] De Caterina R, Cipollone F, Filardo FP, et al. Low-density lipoprotein level reduction by the 3-hydroxy-3-methylglutaryl coenzyme-A inhibitor simvastatin is accompanied by a related reduction of F2-isoprostane formation in hypercholesterolemic subjects: no further effect of vitamin E. Circulation. 2002;106(20):2543–2549. 12427649 10.1161/01.cir.0000038500.43292.d7

[60] Jula A, Marniemi J, Huupponen R, et al. Effects of diet and simvastatin on serum lipids, insulin, and antioxidants in hypercholesterolemic men: a randomized controlled trial. JAMA. 2002;287(5):598–605. 11829698 10.1001/jama.287.5.598

[61] Pereira EC, Bertolami MC, Faludi AA, et al. Antioxidant effect of simvastatin is not enhanced by its association with alpha-tocopherol in hypercholesterolemic patients. Free Radic Biol Med. 2004;37(9):1440–1448. 15454283 10.1016/j.freeradbiomed.2004.07.019

[62] Shin MJ, Cho EY, Jang Y, et al. A beneficial effect of simvastatin on DNA damage in 242 T allele of the NADPH oxidase p22phox in hypercholesterolemic patients. Clin Chim Acta. 2005;360(1–2):46–51. 15936011 10.1016/j.cccn.2005.04.001

[63] Shin MJ, Chung N, Lee JH, et al. Effects of simvastatin on plasma antioxidant status and vitamins in hypercholesterolemic patients. Int J Cardiol. 2007;118(2):173–177. 17005272 10.1016/j.ijcard.2006.03.089

[64] Manfredini V, Biancini GB, Vanzin CS, et al. Simvastatin treatment prevents oxidative damage to DNA in whole blood leukocytes of dyslipidemic type 2 diabetic patients. Cell Biochem Funct. 2010;28(5):360–366. 20589733 10.1002/cbf.1654

[65] Oranje WA, Sels JP, Rondas-Colbers GJ, et al. Effect of atorvastatin on LDL oxidation and antioxidants in normocholesterolemic type 2 diabetic patients. Clin Chim Acta. 2001;311(2):91–94. 11566168 10.1016/s0009-8981(01)00549-6

[66] Shishehbor MH, Aviles RJ, Brennan ML, et al. Association of nitrotyrosine levels with cardiovascular disease and modulation by statin therapy. JAMA. 2003;289(13):1675–1680. 12672736 10.1001/jama.289.13.1675

[67] Save V, Patil N, Moulik N, et al. Effect of atorvastatin on type 2 diabetic dyslipidemia. J Cardiovasc Pharmacol Ther. 2006;11(4):262–270. 17220473 10.1177/1074248406295523

[68] Tousoulis D, Antoniades C, Vasiliadou C, et al. Effects of atorvastatin and vitamin C on forearm hyperaemic blood flow, asymmetrical dimethylarginine levels and the inflammatory process in patients with type 2 diabetes mellitus. Heart. 2007;93(2):244–246. 16914485 10.1136/hrt.2006.093112 PMC1861391

[69] Cangemi R, Loffredo L, Carnevale R, et al. Early decrease of oxidative stress by atorvastatin in hypercholesterolaemic patients: effect on circulating vitamin E. Eur Heart J. 2008;29(1):54–62. 18065424 10.1093/eurheartj/ehm565

[70] Usharani P, Mateen AA, Naidu MU, et al. Effect of NCB-02, atorvastatin and placebo on endothelial function, oxidative stress and inflammatory markers in patients with type 2 diabetes mellitus: a randomized, parallel-group, placebo-controlled, 8-week study. Drugs R D. 2008;9(4):243–250. 18588355 10.2165/00126839-200809040-00004

[71] Hodgson JM, Watts GF, Playford DA, et al. Coenzyme Q10 improves blood pressure and glycaemic control: a controlled trial in subjects with type 2 diabetes. Eur J Clin Nutr. 2002;56(11):1137–1142. 12428181 10.1038/sj.ejcn.1601464

[72] Tkac I, Molcanyiova A, Javorsky M, et al. Fenofibrate treatment reduces circulating conjugated diene level and increases glutathione peroxidase activity. Pharmacol Res. 2006;53(3):261–264. 16420979 10.1016/j.phrs.2005.12.002

[73] Castano G, Menendez R, Mas R, et al. Effects of policosanol and lovastatin on lipid profile and lipid peroxidation in patients with dyslipidemia associated with type 2 diabetes mellitus. Int J Clin Pharmacol Res. 2002;22(3–4):89–99. 12837046

[74] Mastalerz-Migas A, Reksa D, Pokorski M, et al. Comparison of a statin vs. hypolipemic diet on the oxidant status in hemodialyzed patients with chronic renal failure. J Physiol Pharmacol. 2007;58(Suppl 5(Pt 1)):363–370. 18204148

[75] Guan JZ, Murakami H, Yamato K, et al. Effects of fluvastatin in type 2 diabetic patients with hyperlipidemia: reduction in cholesterol oxidation products and VCAM-1. J Atheroscler Thromb. 2004;11(2):56–61. 15153664 10.5551/jat.11.56

[76] Balogh Z, Seres I, Harangi M, et al. Gemfibrozil increases paraoxonase activity in type 2 diabetic patients. A new hypothesis of the beneficial action of fibrates? Diabetes Metab. 2001;27(5 Pt 1):604–610. 11694861

[77] Koh KK, Son JW, Ahn JY, et al. Simvastatin combined with ramipril treatment in hypercholesterolemic patients. Hypertension. 2004;44(2):180–185. 15184351 10.1161/01.HYP.0000133310.42762.25

[78] Skrha J, Stulc T, Hilgertova J, et al. Effect of simvastatin and fenofibrate on endothelium in type 2 diabetes. Eur J Pharmacol. 2004;493(1–3):183–189. 15189781 10.1016/j.ejphar.2004.04.025

[79] Seber S, Ucak S, Basat O, et al. The effect of dual PPAR alpha/gamma stimulation with combination of rosiglitazone and fenofibrate on metabolic parameters in type 2 diabetic patients. Diabetes Res Clin Pract. 2006;71(1):52–58. 16009445 10.1016/j.diabres.2005.05.009

[80] Werba JP, Cavalca V, Veglia F, et al. A new compound-specific pleiotropic effect of statins: modification of plasma gamma-tocopherol levels. Atherosclerosis. 2007;193(1):229–233. 16860808 10.1016/j.atherosclerosis.2006.06.020

[81] Cangemi R, Loffredo L, Carnevale R, et al. Statins enhance circulating vitamin E. Int J Cardiol. 2008;123(2):172–174. 17306387 10.1016/j.ijcard.2006.11.115

[82] Hogue JC, Lamarche B, Tremblay AJ, et al. Differential effect of atorvastatin and fenofibrate on plasma oxidized low-density lipoprotein, inflammation markers, and cell adhesion molecules in patients with type 2 diabetes mellitus. Metab Clin Exp. 2008;57(3):380–386. 18249211 10.1016/j.metabol.2007.10.014

[83] Koksal M, Eren MA, Turan MN, et al. The effects of atorvastatin and rosuvastatin on oxidative stress in diabetic patients. Eur J Intern Med. 2011;22(3):249–253. 21570643 10.1016/j.ejim.2010.12.003

[84] Khanna HD, Sinha MK, Khanna S, et al. Oxidative stress in hypertension: association with antihypertensive treatment. Indian J Physiol Pharmacol. 2008;52(3):283–287. 19552060

[85] Sorrentino SA, Besler C, Rohrer L, et al. Endothelial-vasoprotective effects of high-density lipoprotein are impaired in patients with type 2 diabetes mellitus but are improved after extended-release niacin therapy. Circulation. 2010;121(1):110–122. 20026785 10.1161/CIRCULATIONAHA.108.836346

[86] Rachmani R, Lidar M, Brosh D, et al. Oxidation of low-density lipoprotein in normotensive type 2 diabetic patients. Comparative effects of enalapril versus nifedipine: a randomized cross-over over study. Diabetes Res Clin Pract. 2000;48(2):139–145. 10802151 10.1016/s0168-8227(99)00149-7

[87] Baykal Y, Yilmaz MI, Celik T, et al. Effects of antihypertensive agents, alpha receptor blockers, beta blockers, angiotensin-converting enzyme inhibitors, angiotensin receptor blockers and calcium channel blockers, on oxidative stress. J Hypertens. 2003;21(6):1207–1211. 12777959 10.1097/00004872-200306000-00022

[88] Bank AJ, Kelly AS, Thelen AM, et al. Effects of carvedilol versus metoprolol on endothelial function and oxidative stress in patients with type 2 diabetes mellitus. Am J Hypertens. 2007;20(7):777–783. 17586413 10.1016/j.amjhyper.2007.01.019

[89] Sawada T, Takahashi T, Yamada H, et al. Rationale and design of the KYOTO HEART study: effects of valsartan on morbidity and mortality in uncontrolled hypertensive patients with high risk of cardiovascular events. J Hum Hypertens. 2009;23(3):188–195. 18800142 10.1038/jhh.2008.116

[90] Lim S, Barter P. Antioxidant effects of statins in the management of cardiometabolic disorders. J Atheroscler Thromb. 2014;21(10):997–1010. 25132378 10.5551/jat.24398

[91] Mason RP. Molecular basis of differences among statins and a comparison with antioxidant vitamins. Am J Cardiol. 2006;98(11A):34–41P. 10.1016/j.amjcard.2006.09.018 17126678

[92] Profumo E, Buttari B, Saso L, et al. Pleiotropic effects of statins in atherosclerotic disease: focus on the antioxidant activity of atorvastatin. Curr Top Med Chem. 2014;14(22):2542–2551. 25478882 10.2174/1568026614666141203130324

[93] Ali F, Hamdulay SS, Kinderlerer AR, et al. Statin-mediated cytoprotection of human vascular endothelial cells: a role for Kruppel-like factor 2-dependent induction of heme oxygenase-1. J Thromb Haemost. 2007;5(12):2537–2546. 17927807 10.1111/j.1538-7836.2007.02787.x

[94] Vecchione C, Gentile MT, Aretini A, et al. A novel mechanism of action for statins against diabetes-induced oxidative stress. Diabetologia. 2007;50(4):874–880. 17279352 10.1007/s00125-007-0597-0

[95] Kei A, Tellis C, Liberopoulos E, et al. Effect of switch to the highest dose of rosuvastatin versus add-on-statin fenofibrate versus add-on-statin nicotinic acid/laropiprant on oxidative stress markers in patients with mixed dyslipidemia. Cardiovasc Ther. 2014;32(4):139–146. 24618208 10.1111/1755-5922.12072

[96] Emami-Riedmaier A, Schaeffeler E, Nies AT, et al. Stratified medicine for the use of antidiabetic medication in treatment of type II diabetes and cancer: where do we go from here? J Intern Med. 2015;277(2):235–247. 25418285 10.1111/joim.12330

[97] Viollet B, Guigas B, Sanz Garcia N, et al. Cellular and molecular mechanisms of metformin: an overview. Clin Sci. 2012;122(6):253–270. 22117616 10.1042/CS20110386 PMC3398862

[98] Bonnefont-Rousselot D, Raji B, Walrand S, et al. An intracellular modulation of free radical production could contribute to the beneficial effects of metformin towards oxidative stress. Metab Clin Exp. 2003;52(5):586–589. 12759888 10.1053/meta.2003.50093

[99] Ouslimani N, Peynet J, Bonnefont-Rousselot D, et al. Metformin decreases intracellular production of reactive oxygen species in aortic endothelial cells. Metab Clin Exp. 2005;54(6):829–834. 15931622 10.1016/j.metabol.2005.01.029

[100] Bagi Z, Koller A, Kaley G. PPARgamma activation, by reducing oxidative stress, increases NO bioavailability in coronary arterioles of mice with Type 2 diabetes. Am J Physiol Heart Circ Physiol. 2004;286(2):H742–H748. 14551045 10.1152/ajpheart.00718.2003

[101] Boekholdt SM, Meuwese MC, Day NE, et al. Plasma concentrations of ascorbic acid and C-reactive protein, and risk of future coronary artery disease, in apparently healthy men and women: the EPIC-Norfolk prospective population study. Br J Nutr. 2006;96(3):516–522. 16925857

[102] Hirsch IB, Evans TC, Gaster B. Insulin treatment for type 2 diabetes. JAMA. 1998;279(19):1524–1526. 9605889

[103] Ma SW, Benzie IF, Chu TT, et al. Effect of Panax ginseng supplementation on biomarkers of glucose tolerance, antioxidant status and oxidative stress in type 2 diabetic subjects: results of a placebo-controlled human intervention trial. Diabetes Obes Metab. 2008;10(11):1125–1127. 18355331 10.1111/j.1463-1326.2008.00858.x

[104] Reddy VS, Agrawal P, Sethi S, et al. Associations of FPG, A1C and disease duration with protein markers of oxidative damage and antioxidative defense in type 2 diabetes and diabetic retinopathy. Eye. 2015;29(12):1585–1593. 26381098 10.1038/eye.2015.177 PMC5129785

[105] Sargeant LA, Wareham NJ, Bingham S, et al. Vitamin C and hyperglycemia in the European Prospective Investigation into Cancer – Norfolk (EPIC-Norfolk) study: a population-based study. Diabetes Care. 2000;23(6):726–732. 10840986 10.2337/diacare.23.6.726

[106] Seghrouchni I, Drai J, Bannier E, et al. Oxidative stress parameters in type I, type II and insulin-treated type 2 diabetes mellitus; insulin treatment efficiency. Clin Chim Acta. 2002;321(1–2):89–96. 12031597 10.1016/s0009-8981(02)00099-2

[107] Yamada H, Yamada K, Waki M, et al. Lymphocyte and plasma vitamin C levels in type 2 diabetic patients with and without diabetes complications. Diabetes Care. 2004;27(10):2491–2492. 15451922 10.2337/diacare.27.10.2491

[108] Jiménez-Osorio AS, Picazo A, González-Reyes S, et al Nrf2 and redox status in prediabetic and diabetic patients. Int J Mol Sci. 2014;15(11):20290–20305. 25383674 10.3390/ijms151120290 PMC4264167

[109] Al-Aubaidy HA, Jelinek HF. Oxidative DNA damage: antioxidant response in postprandial hyperglycemia in type 2 diabetes mellitus. Br J Diab Vasc Dis. 2011;11:87–91.

[110] Mahboob M, Rahman MF, Grover P. Serum lipid peroxidation and antioxidant enzyme levels in male and female diabetic patients. Singapore Med J. 2005;46(7):322–324.15968442

[111] Faure P, Wiernsperger N, Polge C, et al. Impairment of the antioxidant properties of serum albumin in patients with diabetes: protective effects of metformin. Clin Sci. 2008;114(3):251–256. 17922677 10.1042/CS20070276

[112] Turk HM, Sevinc A, Camci C, et al. Plasma lipid peroxidation products and antioxidant enzyme activities in patients with type 2 diabetes mellitus. Acta Diabetol. 2002;39(3):117–122. 12357295 10.1007/s005920200029

[113] Shin CS, Moon BS, Park KS, et al. Serum 8-hydroxy-guanine levels are increased in diabetic patients. Diabetes Care. 2001;24(4):733–737. 11315839 10.2337/diacare.24.4.733

[114] Rehman A, Nourooz-Zadeh J, Moller W, et al. Increased oxidative damage to all DNA bases in patients with type II diabetes mellitus. FEBS Lett. 1999;448(1):120–122. 10217422 10.1016/s0014-5793(99)00339-7

[115] Stringer DM, Sellers EA, Burr LL, et al. Altered plasma adipokines and markers of oxidative stress suggest increased risk of cardiovascular disease in First Nation youth with obesity or type 2 diabetes mellitus. Pediatr Diabetes. 2009;10(4):269–277. 19175895 10.1111/j.1399-5448.2008.00473.x

[116] Nakanishi S, Suzuki G, Kusunoki Y, et al. Increasing of oxidative stress from mitochondria in type 2 diabetic patients. Diabetes Metab Res Rev. 2004;20(5):399–404. 15343586 10.1002/dmrr.469

[117] Pan HZ, Zhang H, Chang D, et al. The change of oxidative stress products in diabetes mellitus and diabetic retinopathy. Br J Ophthalmol. 2008;92(4):548–551. 18369071 10.1136/bjo.2007.130542

[118] Kalaivanam KN, Dharmalingam M, Marcus SR. Lipid peroxidation in type 2 diabetes mellitus. Int J Diab Dev Ctries. 2006;26(1):30–32.

[119] Ramakrishna V, Jailkhani R. Oxidative stress in non-insulin-dependent diabetes mellitus (NIDDM) patients. Acta Diabetol. 2008;45(1):41–46. 17924055 10.1007/s00592-007-0018-3

[120] Giaccari A, Sorice G, Muscogiuri G. Glucose toxicity: the leading actor in the pathogenesis and clinical history of type 2 diabetes – mechanisms and potentials for treatment. Nutr Metab Cardiovasc Dis. 2009;19(5):365–377. 19428228 10.1016/j.numecd.2009.03.018

[121] Monnier L, Colette C, Owens DR. Integrating glycaemic variability in the glycaemic disorders of type 2 diabetes: a move towards a unified glucose tetrad concept. Diabetes Metab Res Rev. 2009;25(5):393–402. 19437415 10.1002/dmrr.962

[122] Quagliaro L, Piconi L, Assaloni R, et al. Intermittent high glucose enhances apoptosis related to oxidative stress in human umbilical vein endothelial cells: the role of protein kinase C and NAD(P)H-oxidase activation. Diabetes. 2003;52(11):2795–2804. 14578299 10.2337/diabetes.52.11.2795

[123] Choi SW, Benzie IF, Ma SW, et al. Acute hyperglycemia and oxidative stress: direct cause and effect? Free Radic Biol Med. 2008;44(7):1217–1231. 18226604 10.1016/j.freeradbiomed.2007.12.005

[124] Hirsch IB, Brownlee M. Should minimal blood glucose variability become the gold standard of glycemic control? J Diabetes Complications. 2005;19(3):178–181. 15866065 10.1016/j.jdiacomp.2004.10.001

[125] Giacco F, Brownlee M. Oxidative stress and diabetic complications. Circ Res. 2010;107(9):1058–1070. 21030723 10.1161/CIRCRESAHA.110.223545 PMC2996922

[126] Matough FA, Budin SB, Hamid ZA, et al. The role of oxidative stress and antioxidants in diabetic complications. Sultan Qaboos Univ Med J. 2012;12(1):5–18. 22375253 10.12816/0003082 PMC3286717

[127] Ward NC, Wu JH, Clarke MW, et al. The effect of vitamin E on blood pressure in individuals with type 2 diabetes: a randomized, double-blind, placebo-controlled trial. J Hypertens. 2007;25(1):227–234. 17143195 10.1097/01.hjh.0000254373.96111.43

[128] Musiek ES, Morrow JD. F2-isoprostanes as markers of oxidant stress: an overview. Curr Protoc Toxicol. 2005;10S1:S10–S23. 10.1002/0471140856.tx1705s24 23045114

[129] Tessier DM, Khalil A, Trottier L, et al. Effects of vitamin C supplementation on antioxidants and lipid peroxidation markers in elderly subjects with type 2 diabetes. Arch Gerontol Geriatr. 2009;48(1):67–72. 18077012 10.1016/j.archger.2007.10.005

[130] Erden-Inal M, Sunal E, Kanbak G. Age-related changes in the glutathione redox system. Cell Biochem Funct. 2002;20(1):61–66. 11835271 10.1002/cbf.937

[131] Nuttall SL MU, Kendall MJ, Dunne F. Short-term antioxidant supplementation reduces oxidative stress in elderly patients with type 2 diabetes mellitus a pilot study. Pract Diabetes. 2002;19(7):199–202.

[132] Gazis A, White DJ, Page SR, et al. Effect of oral vitamin E (alpha-tocopherol) supplementation on vascular endothelial function in Type 2 diabetes mellitus. Diabet Med. 1999;16(4):304–311. 10220204 10.1046/j.1464-5491.1999.00049.x

[133] Wu JH, Ward NC, Indrawan AP, et al. Effects of alpha-tocopherol and mixed tocopherol supplementation on markers of oxidative stress and inflammation in type 2 diabetes. Clin Chem. 2007;53(3):511–519. 17272491 10.1373/clinchem.2006.076992

[134] Ble-Castillo JL, Carmona-Diaz E, Mendez JD, et al. Effect of alpha-tocopherol on the metabolic control and oxidative stress in female type 2 diabetics. Biomed Pharmacother. 2005;59(6):290–295. 15932790 10.1016/j.biopha.2005.05.002

[135] Levine M, Conry-Cantilena C, Yet al W. Vitamin C pharmacokinetics in healthy volunteers: evidence for a recommended dietary allowance. PNAS. 1996;93(8):3704–3709. 8623000 10.1073/pnas.93.8.3704 PMC39676

[136] Levine M, Rumsey SC, Daruwala R, et al. Criteria and recommendations for vitamin C intake. JAMA. 1999;281(15):1415–1423. 10217058 10.1001/jama.281.15.1415

[137] Horwitt MK. Critique of the requirement for vitamin E. Am J Clin Nutr. 2001;73(6):1003–1005. 11382651 10.1093/ajcn/73.6.1003

[138] Anderson JW, Gowri MS, Turner J, et al. Antioxidant supplementation effects on low-density lipoprotein oxidation for individuals with type 2 diabetes mellitus. J Am Coll Nutr. 1999;18(5):451–461. 10511327 10.1080/07315724.1999.10718883

[139] Devaraj S, Jialal I. Alpha tocopherol supplementation decreases serum C-reactive protein and monocyte interleukin-6 levels in normal volunteers and type 2 diabetic patients. Free Radic Biol Med. 2000;29(8):790–792. 11053781 10.1016/s0891-5849(00)00420-2

[140] Darko D, Dornhorst A, Kelly FJ, et al. Lack of effect of oral vitamin C on blood pressure, oxidative stress and endothelial function in Type II diabetes. Clin Sci. 2002;103(4):339–344. 12241530 10.1042/cs1030339

[141] Mazloom Z, Hejazi N, Dabbaghmanesh MH, et al. Effect of vitamin C supplementation on postprandial oxidative stress and lipid profile in type 2 diabetic patients. Pakistan J Biol Sci: PJBS. 2011;14(19):900–904. 10.3923/pjbs.2011.900.904 22518934

[142] Shab-Bidar S, Mazloum Z, Mousavi-Shirazifard Z. Daily vitamin E supplementation does not improve metabolic and glycemic control in type 2 diabetic patients: a double blinded randomized controlled trial. J Diabetes. 2013;5(1):57–58. 22607054 10.1111/j.1753-0407.2012.00206.x

[143] Lykkesfeldt J, Poulsen HE. Is vitamin C supplementation beneficial? Lessons learned from randomised controlled trials. Br J Nutr. 2010;103(9):1251–1259. 20003627 10.1017/S0007114509993229

[144] Beckman JA, Goldfine AB, Gordon MB, et al. Oral antioxidant therapy improves endothelial function in Type 1 but not Type 2 diabetes mellitus. Am J Physiol Heart Circ Physiol. 2003;285(6):H2392–H2398. 12881209 10.1152/ajpheart.00403.2003

[145] Evans P, Halliwell B. Micronutrients: oxidant/antioxidant status. Br J Nutr. 2001;85(Suppl2):S67–74. 11509092

[146] McGavin JK, Perry CM, Goa KL. Gliclazide modified release. Drugs. 2002;62(9):1357–1364. 12076188 10.2165/00003495-200262090-00010

[147] O'Brien RC, Luo M, Balazs N, et al. In vitro and in vivo antioxidant properties of gliclazide. J Diab Compl. 2000;14(4):201–206. 10.1016/s1056-8727(00)00084-2 11004429

[148] Prato S D, Pulizzi N. The place of sulfonylureas in the therapy for type 2 diabetes mellitus. Metab Clin Exp. 2006;55(5Suppl1):S20–S27. 10.1016/j.metabol.2006.02.003 16631807

[149] Girnun GD, Domann FE, Moore SA, et al. Identification of a functional peroxisome proliferator-activated receptor response element in the rat catalase promoter. Mol Endocrinol. 2002;16(12):2793–2801. 12456800 10.1210/me.2002-0020

[150] Inoue I, Goto S, Matsunaga T, et al. The ligands/activators for peroxisome proliferator-activated receptor alpha (PPARalpha) and PPARgamma increase Cu2+,Zn2+-superoxide dismutase and decrease p22phox message expressions in primary endothelial cells. Metab Clin Exp. 2001;50(1):3–11. 11172467 10.1053/meta.2001.19415

[151] Khoo NK, Hebbar S, Zhao W, et al. Differential activation of catalase expression and activity by PPAR agonists: implications for astrocyte protection in anti-glioma therapy. Redox Biol. 2013;1:70–79. 24024139 10.1016/j.redox.2012.12.006 PMC3757675

[152] Chung CJ, Pu YS, Chen YT, et al. Protective effects of plasma alpha-tocopherols on the risk of inorganic arsenic-related urothelial carcinoma. Sci Total Environ. 2011;409(6):1039–1045. 21227482 10.1016/j.scitotenv.2010.11.037

[153] Galli A, Mello T, Ceni E, et al. The potential of antidiabetic thiazolidinediones for anticancer therapy. Expert Opin Investig Drugs. 2006;15(9):1039–1049. 10.1517/13543784.15.9.1039 16916271

[154] Ros R D, Assaloni R, Ceriello A. The preventive anti-oxidant action of thiazolidinediones: a new therapeutic prospect in diabetes and insulin resistance. Diabet Med. 2004;21(11):1249–1252. 15498094 10.1111/j.1464-5491.2004.01312.x

[155] Khouri H, Collin F, Bonnefont-Rousselot D, et al. Radical-induced oxidation of metformin. Eur J Biochem. 2004;271(23–24):4745–4752. 15606761 10.1111/j.1432-1033.2004.04438.x

[156] Ruggiero-Lopez D, Lecomte M, Moinet G, et al. Reaction of metformin with dicarbonyl compounds. possible implication in the inhibition of advanced glycation end product formation. Biochem Pharmacol. 1999;58(11):1765–1773. 10571251 10.1016/s0006-2952(99)00263-4

[157] Gallo A, Ceolotto G, Pinton P, et al. Metformin prevents glucose-induced protein kinase C-beta2 activation in human umbilical vein endothelial cells through an antioxidant mechanism. Diabetes. 2005;54(4):1123–1131. 15793252 10.2337/diabetes.54.4.1123

[158] Pagano PJ, Chanock SJ, Siwik DA, et al. Angiotensin II induces p67phox mRNA expression and NADPH oxidase superoxide generation in rabbit aortic adventitial fibroblasts. Hypertension. 1998;32(2):331–337. 9719063 10.1161/01.hyp.32.2.331

[159] Wassmann S, Laufs U, Muller K, et al. Cellular antioxidant effects of atorvastatin in vitro and in vivo. Arterioscler Thromb Vasc Biol. 2002;22(2):300–305. 11834532 10.1161/hq0202.104081

[160] Takemoto M, Node K, Nakagami H, et al. Statins as antioxidant therapy for preventing cardiac myocyte hypertrophy. J Clin Invest. 2001;108(10):1429–1437. 11714734 10.1172/JCI13350 PMC209420

